# Melissa: Bayesian clustering and imputation of single-cell methylomes

**DOI:** 10.1186/s13059-019-1665-8

**Published:** 2019-03-21

**Authors:** Chantriolnt-Andreas Kapourani, Guido Sanguinetti

**Affiliations:** 1 School of Informatics, University of Edinburgh, Edinburgh, EH8 9AB UK; 2 MRC Institute of Genetics and Molecular Medicine, University of Edinburgh, Edinburgh, EH4 2XU UK; 3 Synthetic and Systems Biology, University of Edinburgh, Edinburgh, EH9 3BF UK

## Abstract

Measurements of single-cell methylation are revolutionizing our understanding of epigenetic control of gene expression, yet the intrinsic data sparsity limits the scope for quantitative analysis of such data. Here, we introduce Melissa (MEthyLation Inference for Single cell Analysis), a Bayesian hierarchical method to cluster cells based on local methylation patterns, discovering patterns of epigenetic variability between cells. The clustering also acts as an effective regularization for data imputation on unassayed CpG sites, enabling transfer of information between individual cells. We show both on simulated and real data sets that Melissa provides accurate and biologically meaningful clusterings and state-of-the-art imputation performance.

**Electronic supplementary material:**

The online version of this article (10.1186/s13059-019-1665-8) contains supplementary material, which is available to authorized users.

## Background

DNA methylation is probably the best studied epigenomic mark, due to its well-established heritability and widespread association with diseases and a broad range of biological processes, including X-chromosome inactivation, cell differentiation, and cancer progression [1–3]. Yet its role in gene regulation, and the molecular mechanisms underpinning its association with diseases, is still imperfectly understood.

Bisulfite treatment of DNA followed by sequencing (BS-seq) has provided a powerful tool for measuring the methylation level of cytosines on a genome-wide scale with single nucleotide resolution [4]. BS-seq protocols have been vastly improved over the last decade, with BS-seq rapidly becoming a widespread tool in biomedical investigation. Nevertheless, until very recently, BS-seq could only be used to measure methylation in bulk populations of cells [5], preventing effective investigations of the role of DNA methylation in shaping transcriptional variability and early development [6, 7].

This shortcoming has been addressed within the last 5 years through the development of protocols to measure DNA methylation at single-cell resolution using either scBS-seq [8] or scRRBS [9] making it possible to uncover the heterogeneity and dynamics of DNA methylation [10]. Even more recently, methods have been developed that can sequence both the methylome and the transcriptome or other features in parallel, potentially enabling a quantification of the role of DNA methylation in explaining transcriptional heterogeneity [11–13]. However, due to the small amounts of genomic DNA per cell, these protocols usually result in very sparse genome-wide CpG coverage (i.e., for most CpGs, we have missing values), ranging from 5% in high-throughput studies [14, 15] to 20% in low-throughput ones [8, 11]. The sparsity of the data represents a major hurdle to effectively use single-cell methylation assays to inform our understanding of epigenetic control of transcriptomic variability, or to distinguish individual cells based on their epigenomic state.

In this paper, we address these problems by using a two-pronged strategy. First, we note that several recent studies have highlighted the importance of local methylation profiles, as opposed to individual CpG methylation, in determining the epigenetic state of a region [16–18]. This implies that local spatial correlations may be effectively leveraged to ameliorate the issue of data sparsity. Secondly, single-cell BS-seq protocols, as all single-cell high-throughput protocols, simultaneously assay a large number of cells, ranging from several tens [8] to a few thousands in the most recent studies [14]. Such abundance of data could be exploited to our advantage to transfer information across similar cells.

We implement both of these strategies within Melissa (MEthyLation Inference for Single cell Analysis), a Bayesian hierarchical model that jointly learns the methylation profiles of genomic regions of interest and clusters cells based on their genome-wide methylation patterns. In this way, Melissa can effectively use both the information of neighboring CpGs and of other cells with similar methylation patterns in order to predict CpG methylation states. As an additional benefit, Melissa also provides a Bayesian clustering approach capable of identifying subsets of cells based solely on epigenetic state, to our knowledge the first clustering method tailored specifically to this rapidly expanding technology. We benchmark Melissa on both simulated and real single-cell BS-seq data, demonstrating that Melissa provides both state-of-the art imputation performance and accurate clustering of cells. Furthermore, thanks to a fast variational Bayes estimation strategy, Melissa has good scalability and can provide an effective modeling tool for the increasingly large single-cell methylation studies which will become prevalent in coming years.

## Results and discussion

**Fig. 1:**
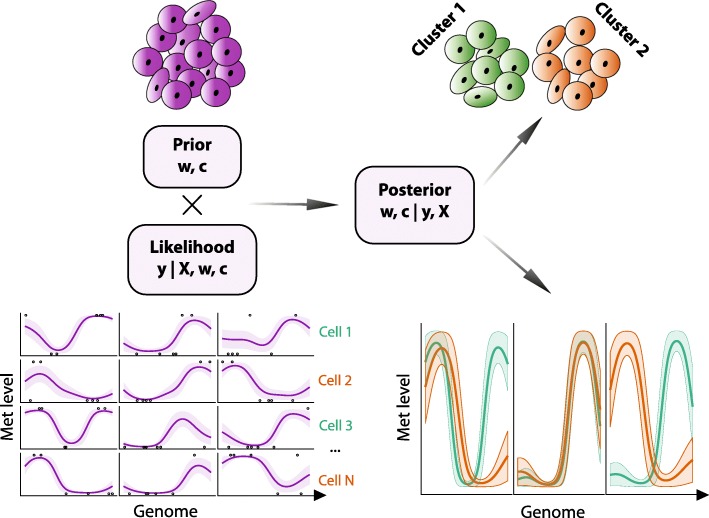
Melissa model overview. Melissa combines a likelihood computed from single-cell methylation profiles fitted to each genomic region using a supervised regression approach (bottom left) and an unsupervised Bayesian clustering prior (top left). The posterior distribution provides a methylome-based clustering (top right) and imputation (bottom right) of single cells

Melissa addresses the data sparsity issue by leveraging local correlations between neighboring CpGs and similarity between individual cells (see Fig. 1). The starting point is the definition of a set of genomic regions (e.g. genes or enhancers) over which the model will be applied. Within each region, Melissa postulates a latent profile of methylation, a function mapping each CpG within the region to a number in [0,1] which defines the probability of that CpG being methylated. To ensure spatial smoothness of the profile, Melissa uses a generalized linear model (GLM) of basis function regression along the lines of [16] (with modified likelihood to account for single cell data). Local correlations are however often insufficient for regions with extremely sparse coverage, and these are quite common in scBS-seq data. Therefore, we share information across different cells by coupling the local GLM regressions through a shared prior distribution. In order to respect the (generally unknown) population structure that may be present within the cells assayed, we choose a (finite) Dirichlet mixture model prior. The output of Melissa is therefore twofold: at each genomic region in each cell, we get a predicted profile of methylation, which can be used to impute missing data (i.e., unassayed CpGs). For each cell, we also get a discrete cluster membership probability, providing a methylome-based clustering of cells. This twofold output of Melissa reflects its methodological foundations as a hybrid between a global unsupervised model (Bayesian clustering of methylomes) and a local supervised learning model (GLM regression for every region). In this sense, Melissa is closer to a *mixture of experts* model [19, Chapter 14, Section 5] than a standard mixture model.

### Benchmarking Melissa on simulated data

We benchmark the ability of our model to cluster and impute CpG methylation states at the single-cell level both on simulated and mouse embryonic stem cell (ESC) data sets. To assess test prediction performance, we consider different metrics, including *F*-measure, the area under the receiver operating characteristic curve (AUC), and precision recall curves [20]. We explore the performance of a number of methods as we vary three possible experimental parameters: the number of cells assayed, the cluster dissimilarity (how different the methylomes of cells in different clusters are expected to be), and the CpG coverage (defined as the fraction of CpG sites covered by at least one read, averaged over all cells).

To benchmark the performance of Melissa in predicting CpG methylation states, we compare it against six different imputation strategies. As a baseline approach, we compute the average methylation rate separately for each cell and region (*Rate*), that is, the average is taken over all CpG sites forming a genomic region. We also use the BPRMeth model [16, 21], where we account for the binary nature of the observations, which we train independently across cells and regions (*BPRMeth*). Note that BPRMeth shares information across CpG sites inside each genomic region; however, it does not transfer information across cells. To share information across cells, but not across neighboring CpGs inside the region, we constrain Melissa to infer constant functions, i.e., learn average methylation rate (*Melissa rate*). We also use a Gaussian mixture model (*GMM*) that takes as input average *M* values [22] instead of average methylation rates across the region (see the “Methods” section); to avoid possible problems due to high-dimensionality, the GMM method was also tested on reduced-dimensionality data, where the first ten principal components were retained. Additionally, as a fully independent baseline, we use a Random Forest classifier trained on individual cells and regions, where the input features are the observed CpG locations, and the response variable is the CpG methylation state: methylated or unmethylated (*RF*). This is essentially the method of [23], however, without using additional annotation data or DNA sequence patterns. We delay comparisons with the deep learning method *DeepCpG* [24] to the next section, as DeepCpG is not applicable in the settings of this simulation (see below).

In order to generate realistic simulated single-cell DNA methylation data, we extracted methylation profiles from real (bulk) BS-seq data using the BPRMeth package [21], and then generated binary methylation levels at a random subset of CpGs to simulate the low coverage of scBS-seq. In total, we simulated *N*=200 cells from *K*=4 sub-populations, where each cell consisted of *M*=100 genomic regions. Additionally, to account for different levels of similarity between cell sub-populations, we simulated 11 different data sets by varying the proportion of similar genomic regions between clusters. Finally, to assess the performance of Melissa as a function of assayed single cells, we simulated 10 different data sets by varying *N*, the total number of single cells (see the “Methods” section).

**Fig. 2:**
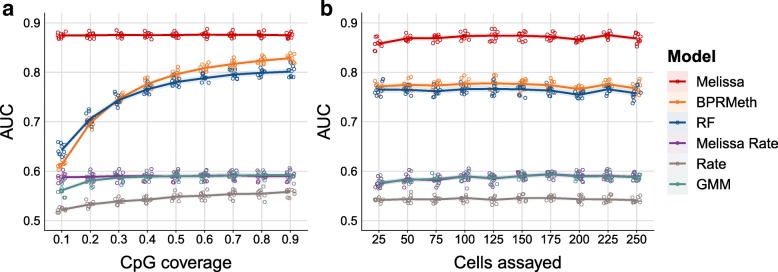
Melissa robustly imputes CpG methylation states. **a** Imputation performance in terms of AUC as we vary the proportion of covered CpGs used for training. Higher values correspond to better imputation performance. For each CpG coverage setting, a total of 10 random splits of the data to training and test sets was performed. Each colored circle corresponds to a different simulation. The plot shows also the LOESS curve for each method as we increase CpG coverage. The methods considered were *Melissa* which shares information across cells and neighboring CpGs, the *BPRMeth* model that only shares information across neighboring CpGs, and a Random Forest classifier (*RF*) which predicts CpG methylation states using as input the observed CpG locations. Additionally, we considered three baseline models: Melissa Rate that transfers information across cells but not across neighboring CpGs using mean methylation levels across the genomic region, a Gaussian mixture model (GMM) that takes as input average *M* values across the region, and finally, the *Rate* method where we compute a mean methylation rate separately for each cell and genomic region. **b** Imputation performance measured by AUC for varying number of cells assayed. In **a**, N = 200 cells were simulated and cluster dissimilarity was set to 0.5, and in **b**, CpG coverage was set to 0.4 and cluster dissimilarity to 0.5

Applying the competing methods to synthetic data, we observe that Melissa yields a substantial improvement in prediction accuracy compared to all other models (Fig. 2, Additional file 1: Figure S1 and S2). Notably, Melissa is robust across different settings of the data, such as CpG coverage proportion (Fig. 2a) or the total number of cells assayed in each experiment (Fig. 2b). Due to its ability to transfer information across cells and neighboring CpGs, our model robustly maintains its prediction accuracy at a very sparse coverage level of 10% or even when assaying around 25 single cells. The *BPRMeth* and *RF* models perform poorly at low CpG coverage settings, becoming comparable to Melissa when using the majority of the CpGs for training set. Importantly, Melissa still performs better at 90% CpG coverage, demonstrating that the clustering acts as an effective regularization for imputing unassayed CpG sites. As expected, *Melissa Rate* and *GMM* have very similar performance (due to the very similar model structure); for both methods, performance is significantly weaker than *Melissa* across the full range of simulation settings, since they are not expressive enough to capture spatial correlations between CpGs. Using GMM on reduced dimensionality data did not lead to an improvement in performance, either for imputation or clustering (data not shown). Finally, the naive *Rate* method has the worst imputation performance of all methods, by a considerable margin. The imputation performance of all methods is relatively insensitive to the degree of cluster dissimilarity (Additional file 1: Figure S2).

**Fig. 3:**
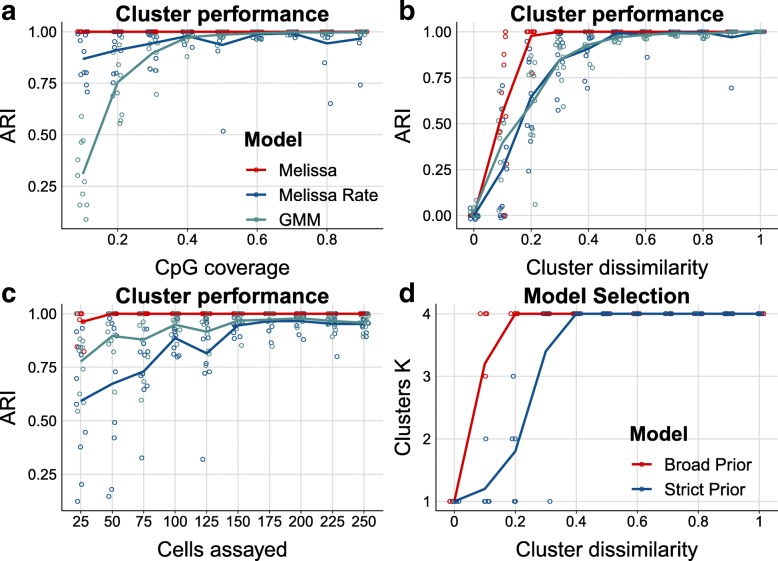
Melissa efficiently and accurately clusters cell sub-populations. **a** Clustering performance measured by ARI as we vary CpG coverage. Higher values correspond to better agreement between predicted and true cluster assignments. For each CpG coverage setting, a total of 10 random splits of the data to training and test sets was performed. Each colored circle corresponds to a different simulation. The plot shows also the LOESS curve for each method as we increase CpG coverage. **b** Clustering performance (ARI) for varying proportions of similar genomic regions between clusters. **c** Clustering performance (ARI) as we vary the total number of cells assayed. **d** Predicted number of clusters using two different prior settings: a broad and a strict prior as we vary cluster dissimilarity. Initial number of clusters was set to *K*=10. Melissa identifies the correct number of clusters in most parameter settings (*K*=4); notably when there is no dissimilarity across clusters (i.e., we have one global cell sub-population), Melissa prunes away all components and keeps only one cluster (*K*=1)

Next, we consider the clustering performance of *Melissa*. Since most of the rival methods do not have a notion of clustering, we compare Melissa to clustering using methylation rates for binary data (Melissa Rate) or Gaussian data (GMM) using *M* values [22]. As a performance metric, we use the Adjusted Rand Index (ARI) [25] between the true cluster assignment and the predicted cluster membership returned from the model. Figure 3a shows ARI values comparing the three models for varying CpG coverage (with cluster dissimilarity level at 0.5 and *N* = 200 cells). Melissa performs perfectly in all settings, demonstrating its power and sensitivity in identifying robustly the cell sub-population structure. When varying the level of cluster dissimilarity (see Fig. 3b), the model is still able to retain its high clustering performance. As expected, for settings with low variability between clusters (i.e., cell sub-populations are difficult to distinguish), the performance drops; however, Melissa is consistently superior to the Melissa Rate and GMM models and rapidly reaches near-perfect clustering accuracy. Similarly, when varying the total number of cells assayed in each experiment (see Fig. 3c), Melissa retains its almost perfect clustering performance and is still consistently superior than the competing models.

Subsequently, we test Melissa’s ability to perform model selection, that is, to identify the appropriate number of cell sub-populations. To do so, we run the model on simulated data, setting the initial number of clusters to *K* = 10 and letting the variational optimization prune away inactive clusters [26]. We used both broad (red line) and shrinkage (blue line) priors. Figure 3d shows that the variational optimization automatically recovered the correct number of mixture components for almost all parameter settings. As expected, in settings with high between cluster similarity, the model with shrinkage prior returned fewer clusters, since the data complexity term in Eq. (9) (see the “Methods” section) was penalizing more the variational approximation compared to the gain in likelihood from explaining the data. Finally, we assess the scalability of Melissa with respect to the number of single cells. Additional file 1: Figure S3 compares the variational Bayes (red line) with the Gibbs sampling (blue line) algorithm, which demonstrates the good scalability of variational inference where we can analyze thousands of single cells in acceptable running times. The maximum number of iterations for the variational Bayes algorithm was set to 400, and the Gibbs algorithm was run for 3000 iterations. Both algorithms are implemented in the R programming language and were run on a machine utilizing at most 16 CPU cores.

### Benchmarking Melissa on sub-sampled bulk ENCODE data

The results in the previous section convincingly showed a substantial advantage of Melissa over competing methods both in terms of imputation performance and in terms of clustering. However, conditioned on some seed profiles learnt from bulk data, the simulation was conducted on data which was directly sampled from the generative Melissa model (with some additional noise), which could conceivably introduce an unfair bias in the comparison. Additionally, since data were simulated as separate regions, comparison with the deep learning method DeepCpG [24] was not possible, since DeepCpG requires the information of a large number of neighboring CpGs to predict the methylation state of each target CpG site. To faithfully simulate scBS-seq data, we generated two additional synthetic data sets by directly sub-sampling bulk ENCODE reduced representation bisulfite sequencing (RRBS) and whole-genome bisulfite sequencing (WGBS) experiments (see the “Methods” section). For the bulk RRBS data, we randomly sub-sampled 10% of the mapped reads and generated 40 pseudo-single cells from the GM12878 and H1-hESC cell lines. Due to the higher sequencing depth of bulk WGBS experiments, only 0.5% of the mapped reads were sub-sampled to generate pseudo-single-cell methylomes. Subsequently, reads falling in the same genomic site were binarised to obtain a digital output of methylation. Finally, the two cell lines were combined in a single data set of 80 pseudo-single cells prior to running Melissa. This procedure produces data with a more similar structure to real scBS-seq data, since the uneven read coverage better captures the structure of missing data observed in single cell epigenomic experiments.

Table 1 shows the results for the two studies when imputing CpGs falling in genomic regions of ± 2.5 kb around transcription start sites (TSS) for different levels of CpG coverage. Consistently with the simulation study in the previous section, Melissa performs significantly better (on scRRBS synethtic data) or comparable (on scWGBS synthetic data) to competitors at imputation tasks. As reported in [24], DeepCpG performs very strongly with comparable accuracy to Melissa across all CpG coverage settings (notice that training of DeepCpG is however slightly different, see “Methods” section). The systematically lower performance of DeepCpG on the scRRBS data set is to be expected as DeepCpG relies on information from neighboring CpGs over a large region, and might therefore be at disadvantage for data generated using this technology. The results are consistent across all different metrics considered in this paper and when increasing the window size to ± 5 kb around TSS (see Additional file 1: Figure S4–S9). Finally, Melissa could easily separate both cell sub-populations for all settings considered in this study.

**Table 1:** Melissa robustly imputes CpG methylation states on sub-sampled ENCODE scRRBS and scWGBS synthetic data. Entries with italics denote the model with the highest performance in terms of AUC

	Pseudo scRRBS		Pseudo scWGBS	
Model	AUC 20% cov	AUC 50% cov	AUC 20% cov	AUC 50% cov
Melissa	*0.96* (7.3×10^−4^)	*0.96* (6.8×10^−4^)	*0.96* (6.3×10^−4^)	*0.96* (6.6×10^−4^)
DeepCpG	0.94 (1.5×10^−3^)	0.94 (1.5×10^−3^)	*0.96* (1.4×10^−3^)	*0.96* (1.4×10^−3^)
BPRMeth	0.88 (2.2×10^−3^)	0.91 (2.5×10^−3^)	0.90 (1.9×10^−3^)	0.92 (1.5×10^−3^)
RF	0.79 (3.2×10^−3^)	0.87 (2.0×10^−3^)	0.83 (2.2×10^−3^)	0.89 (2.1×10^−3^)
Melissa rate	0.88 (1.8×10^−3^)	0.88 (1.3×10^−3^)	0.70 (2.2×10^−3^)	0.71 (2.5×10^−3^)
Rate	0.82 (2.6×10^−3^)	0.84 (2.5×10^−3^)	0.76 (4.2×10^−3^)	0.77 (3.0×10^−3^)

Imputation performance in terms of AUC as we vary the proportion of covered CpGs used for training. Higher values correspond to better imputation performance. For each CpG coverage setting, a total of 10 random splits of the data to training and test sets was performed; shown are the mean AUC value together with two standard deviations of the estimate in parenthesis. Note that DeepCpG was trained once on two chromosomes; hence, the values do not change as we vary the CpG coverage

### Melissa accurately predicts methylation states on real data

To assess Melissa’s performance on real scBS-seq data, we considered two mouse ESC data sets generated from scM&T-seq [11] and scBS-seq [8] protocols. The mouse ESCs were cultured either in 2i medium (*2i ESCs*) or serum conditions (*serum ESCs*); hence, we expect methylation heterogeneity between cell sub-populations. In addition, in *serum ESCs*, there is evidence of additional CpG methylation heterogeneity [27], making these data suitable for the model selection task to infer cell sub-population structure. The analysis on both data sets was performed on six different genomic contexts: protein coding promoters with varying genomic windows: ± 1.5 kb, ± 2.5 kb, and ± 5 kb around TSS, active enhancers, super enhancers, and Nanog regulatory regions (see the “Methods” section for details on data preprocessing). It should be noted that *DeepCpG* is designed to predict individual missing CpGs, rather than missing regions, and requires always information about neighboring CpGs. This means that, during prediction, *DeepCpG* always has access to more data than competing methods, potentially providing it with an unfair advantage; to partly address this problem, we also present results when DeepCpG had access to sub-sampled data (labeled *DeepCpG Sub* in our figures). In general, *DeepCpG* should be thought as complementary to Melissa, and comparisons should be evaluated cautiously (see below).

**Fig. 4:**
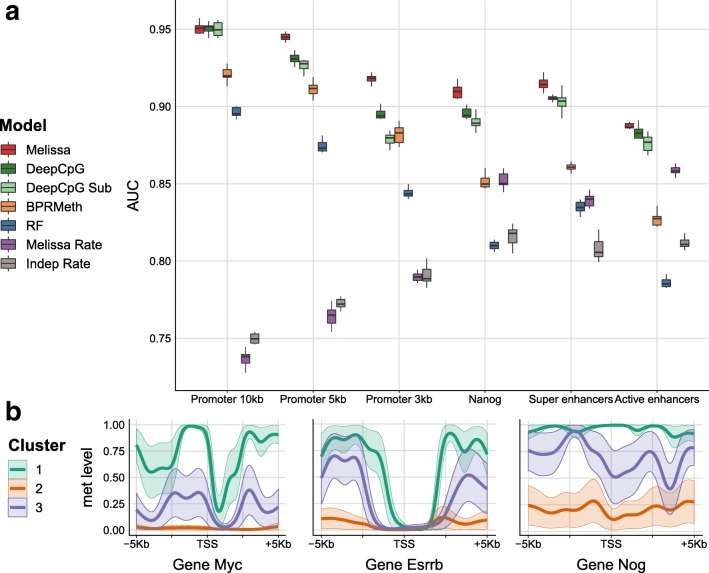
Imputation performance and clustering of scM&T-seq mouse ESCs [11] based on genome wide methylation profiles. **a** Prediction performance on test set for imputing CpG methylation states in terms of AUC. Higher values correspond to better imputation performance. Each colored boxplot indicates the performance using 10 random splits of the data in training and test sets; due to high computational costs, DeepCpG was trained only once and the boxplots denote the variability across ten random sub-samplings of the test set. **b** Example promoter regions with the predicted methylation profiles for three developmental genes: *Myc, Esrrb*, and *Nog*. Each colored profile corresponds to the average methylation pattern of the cells assigned to each sub-population, in our case Melissa identified *K*=3 clusters

We first applied Melissa on the scM&T-seq data set which consists of 75 single cells (14 *2i ESCs* and 61 *serum ESCs*). Figure 4a shows a direct comparison of the imputation performance of all the methods across a variety of genomic contexts. Melissa is better or comparable to rival methods in terms of AUC (see Fig. 4a) and substantially more accurate in terms of *F*-measure (Additional file 1: Figure S10), demonstrating its ability to capture local CpG methylation patterns. *DeepCpG* also performs strongly on most genomic regions, indicating that a flexible deep learning method is effective in capturing patterns of methylation. Similar results were obtained by considering different metrics (Additional file 1: Figure S10–S12). Boxplots show performance distributions across 10 independent training/test splits of the data, except for *DeepCpG*, where the high computational costs prevented such investigation. Interestingly, methods based on methylation rates performed poorly at promoters, underlining the importance of methylation profiles in distinguishing epigenetic state near transcription start sites and identifying meaningful cell sub-populations. For all models, the imputation performance (in terms of AUC) at active enhancers was lower, indicating high methylation variability across cells and neighboring CpG sites as shown in [8].

In terms of clustering performance, Melissa confirms that the data supports the existence of a sub-population of serum cells as suggested previously [27], by returning three clusters in almost all contexts. Further insights on the biological significance of the clusters obtained can be gleaned by inspecting the inferred methylation profiles at relevant regions. Figure 4b shows posterior methylation profiles for three developmental genes for each cell sub-population (Additional file 1: Figure S13 shows additional methylation profiles of developmental genes). Each color corresponds to a different cell sub-population, with orange profiles corresponding to *2i ESCs* which are globally hypo-methylated. The green and purple profiles correspond to serum cells, which, as expected, present an increased level of methylation overall. However, Melissa identifies a clear sub-population structure within these serum cells: the purple cluster clearly represents a sub-population of cells which has only incompletely transitioned towards the final differentiated state (high global methylation punctuated by hypo-methylated CpG islands). Interestingly, 2i cells can be easily separated from serum cells based on methylation rate alone, due to the global hypo-methylation of 2i cells; however, the sub-population structure within serum cells appears to be determined by changes in profiles.

**Fig. 5:**
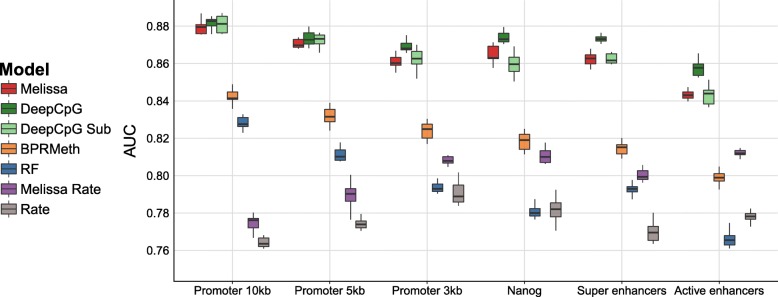
Imputation performance of scBS-seq mouse ESCs [8] based on genome-wide methylation profiles. Shown is the prediction performance, in terms of AUC, for imputing CpG methylation states. Each colored boxplot indicates the performance using 10 random splits of the data in training and test sets; due to high computational costs, DeepCpG was trained only once and the boxplots denote the variability across ten random sub-samplings of the test set

As a second real data set, we analyzed the smaller scBS-seq data set which consists of only 32 cells (12 *2i ESCs* and 20 *serum ESCs*). The imputation performance in terms of AUC across genomic contexts is shown in Fig. 5. Melissa retains its high prediction accuracy and is comparable with DeepCpG across most contexts (see Additional file 1: Figure S14–S16 for performance on different metrics), even though the full DeepCpG model has slightly better performance on this data set. This suggests that the small number of cells in this data set did not allow an effective sharing of information. In terms of clustering performance, Melissa identifies three clusters in the vast majority of settings, once again underlying the emergence of epigenomically distinct populations within serum cells (see Additional file 1: Figure S17 and S18 for example methylation profiles across genomic contexts).

### A note on the comparison with DeepCpG

Melissa and DeepCpG models reported substantially better imputation performance compared to the rival methods and show comparable performance when analyzed on real data sets, demonstrating their flexibility in capturing complex patterns of methylation. However, the two methods have significantly different computational performances. In our experiments, Melissa’s runtime was less than 6 h for all genomic contexts running on a small server machine utilizing at most ten CPU cores (see Additional file 1: Table S1 and S2). By contrast, DeepCpG required around 3 to 4 days to analyze each data set on a GPU cluster equipped with high end NVIDIA Tesla K40ms GPUs, and had very high memory requirements. These computational overheads effectively make DeepCpG out of reach for smaller research groups. On the other hand, Melissa operates on a set of genomic contexts of interest (e.g., promoters), while DeepCpG is designed for genome-wide imputation; computational performance of both methods will therefore depend on specific choices, such as the size/number of the regions of interest for Melissa, or the number of training chromosomes for DeepCpG.

In addition to the differences in scope between the two methods, one should also be cautious when directly comparing prediction performances due to the different design of the DeepCpG model. DeepCpG is trained on a specific set of chromosomes and considers each CpG site independently; hence, it does not have a notion of genomic region to be trained on and will in any case utilize information from neighboring CpGs within or outside the region, information that Melissa and the rival methods do not have access to.

## Conclusions

Single-cell DNA methylation measurements are rapidly becoming a major tool to understand epigenetic gene regulation in individual cells. Newer platforms are rapidly expanding the scope of the technology in terms of assaying large numbers of cells [14]; however, all technologies are plagued by intrinsically low coverage in terms of numbers of CpGs assayed.

In this paper, we have proposed Melissa as a way of addressing the low coverage issue by sharing information between CpGs with a local smoothing and between cells with a Bayesian clustering prior. On both synthetic and real data, Melissa achieved state-of-the art imputation performance over a panel of competing methods, including DeepCpG [24] and random forests. While achieving comparable or superior performance to black-box methods, such as neural networks and random forests, Melissa is more transparent and needs minimal tuning: all the results shown, on both synthetic and real data, were obtained with the same settings of the algorithm. Additionally, as all Bayesian methods, Melissa outputs are probability distributions that fully quantify the uncertainty on the model’s prediction, and which are more easily usable for further experimental design compared to the point-estimates provided by black-box approaches. Melissa does not require additional annotation data as in [23] or [28] and does not exploit sequence information like DeepCpG, but an extension leveraging side data would be easily accomplished within the Bayesian framework and would represent an interesting extension for future research. By using a Bayesian clustering prior, Melissa has the added benefit of simultaneously uncovering the population structure within the assay, as we demonstrated in the real data examples; Melissa can therefore be a useful tool in uncovering epigenetic diversity among cells.

In addition, in this work, Melissa was applied on pre-defined genomic regions of interest, such as promoters and enhancers; however, one could easily perform genome-wide imputation and clustering of single-cell methylomes by using a sliding (non-overlapping) window approach. While this paper was under review, we became aware of a new preprint describing Epiclomal [29], a method to perform clustering of single-cell DNA methylomes using a Bayesian probabilistic model. Epiclomal shares a similar hierarchical structure to Melissa and also models bisulfite conversion error; however, Epiclomal does not model the spatial variability of neighboring CpGs and therefore cannot perform imputation as Melissa does.

While Melissa accounts for heterogeneity in the cell population structure, it does not allow for heterogeneity at the single-gene level: each cluster has a single methylation profile within each region, and all variability at the single locus level is attributed to noise. This rigidity limits the usefulness of Melissa as a tool to investigate intrinsic stochasticity in methylation at the single locus level. Relaxing the modeling assumptions to accommodate methylation variability in Melissa is an interesting topic for future research. Another area where Melissa could be fruitfully applied is the integrative study of multiple high-throughput features in single cells. Recently, Kapourani and Sanguinetti [16] showed that features extracted from methylation profiles could be effectively used to predict gene expression in bulk experiments. With the advent of novel technologies measuring gene expression and multiple epigenomic features in individual cells [13], interpretable Bayesian models like Melissa are likely to play an important role in furthering our understanding of epigenetic control of gene expression in single cells.

## Methods

### Melissa model

In order to provide spatial smoothing of the methylation profiles at specific regions, we adapt a generalized linear model of basis function regression proposed recently [16] and further extended and implemented in the BPRMeth Bioconductor package [21]. The basic idea of BPRMeth is as follows: the methylation profile associated with a genomic region *m* is defined as a (latent) function *f*:*m*→(0,1) which takes as input the genomic coordinate along the region and returns the propensity for that locus to be methylated. For single-cell methylation data, methylation of individual CpG sites can be naturally modeled using a Bernoulli observation model, since for the majority of covered sites we have binary CpG methylation states (see Additional file 1: Figure S13). More specifically, for a specific region *m*, we model the observed methylation of CpG site *i* as:

1 $$ y_{mi} \sim \mathcal{B}\text{ern}(\rho_{mi}),  $$

where the unknown “true” methylation level *ρ*_*mi*_ has as covariates the CpG locations *x*_*mi*_. Then, we define the BPRMeth model as:

2 $$ \begin{aligned} \eta_{mi} & = \mathbf{w}_{m}^{\top}\mathbf{h}(x_{mi}), \\ f_{m}(x_{mi}) & = \rho_{mi} = g^{-1}(\eta_{mi}), \end{aligned}  $$

where **w**_*m*_ are the regression coefficients, **x**_*mi*_≡**h**(*x*_*mi*_) are the basis function transformed CpG locations (here we consider radial basis functions (RBFs)), and *g*(·) is the link function that allows us to move from the systematic components *η*_*mi*_ to mean parameters *ρ*_*mi*_. Here we consider a *probit regression* model which is obtained by defining *g*^−1^(·) = *Φ*(·) — where *Φ*(·) denotes the cdf of the standard normal distribution—ensuring that *f* takes values in the [0,1] interval. Notice that both BPRMeth and Melissa do not explicitly model bisulfite conversion errors. Conversion errors are estimated to be relatively rare and below 1% [30], and we show in our simulation studies that Melissa is highly robust to the addition of noise mimicking possible errors.

**Fig. 6:**
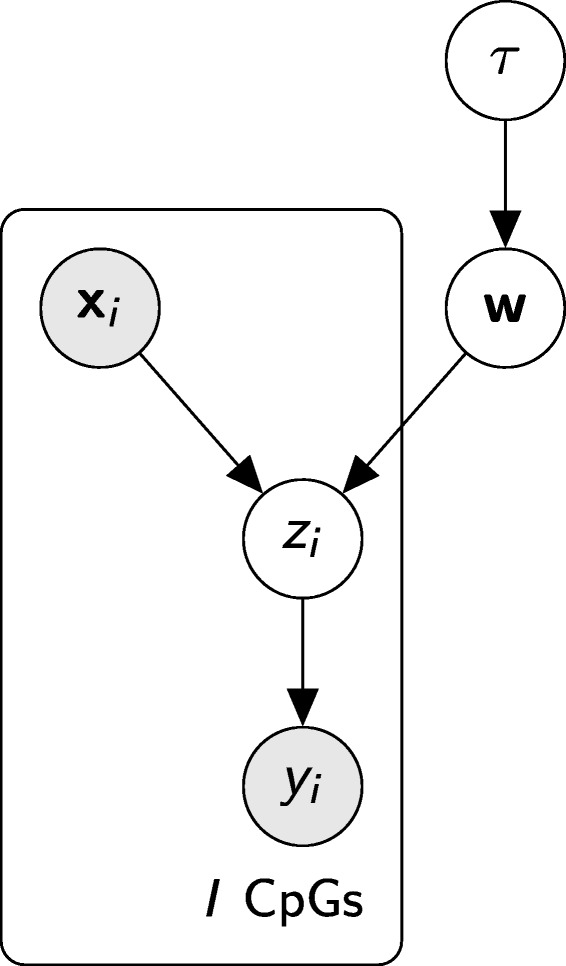
Probabilistic graphical representation of the BPRMeth model

To account for the limited CpG coverage of scBS-seq experiments, the BPRMeth model was recently reformulated in a Bayesian framework [21]. The model was made amenable to Bayesian estimation thanks to a data augmentation strategy [31]. This strategy consists of introducing an additional auxiliary latent variable *z*_*i*_, which has a Gaussian distribution conditioned on the input **w**^⊤^**x**_*i*_, leading to the graphical model in Fig. 6.

The BPRMeth model is limited to sharing informati.on across CpGs via local smoothing (which certainly helps in dealing with data sparsity); however, in our experience the coverage in scBS-seq data is insufficient to infer informative methylation profiles at many genomic regions. We therefore propose Melissa to exploit the population structure of the experimental design and additionally share and transfer information across cells.

Assume that we have *N*(*n*=1,...,*N*) cells and each cell consists of *M*(*m*=1,...,*M*) genomic regions, for example promoters, and we are interested in both partitioning the cells in *K* clusters and inferring the methylation profiles for each genomic region. To do so, we use a finite Dirichlet mixture model (FDMM) [32], where we assume that the methylation profile of the *m*^*th*^ region for each cell *n* is drawn from a mixture distribution with *K* components (where *K*<*N*). This way, cells belonging to the same cluster will share the same methylation profile, although profiles will still differ across genomic regions. Let **c**_*n*_ be a latent variable comprising a 1-of-K binary vector with elements *c*_*nk*_ representing the component that is responsible for cell *n*, and *π*_*k*_ be the probability that a cell belongs to cluster *k*, i.e. *π*_*k*_=*p*(*c*_*nk*_=1). The conditional distribution of **C**={**c**_1_,…,*c*_*N*_} given ***π*** is:

3 $$ p(\mathbf{C} \thinspace | \thinspace \boldsymbol{\pi}) = \prod_{n=1}^{N}\prod_{k=1}^{K}\pi_{k}^{c_{nk}}.  $$

Considering the FDMM as a generative model, the latent variables **c**_*n*_ will generate the latent observations $\mathbf {Z}_{n} \in \mathbb {R}^{M\times I_{m}}$, which in turn will generate the binary observations $\mathbf {Y}_{n} \in \{0, 1\}^{M\times I_{m}}$ depending on the sign of **Z**_*n*_, as shown in Fig. 6. The conditional distribution of the data (**Z**,**Y**), given the latent variables **C** and the component parameters **W**, becomes:

4 $$ {\begin{aligned} p(\mathbf{Y}, \mathbf{Z} \thinspace | \thinspace \mathbf{C}, \mathbf{W}, \mathbf{X}) & = \prod_{n=1}^{N}\prod_{k=1}^{K}\left[\prod_{m=1}^{M}p(\mathbf{y}_{nm} \thinspace | \thinspace \mathbf{z}_{nm}) \thinspace p(\mathbf{z}_{nm} \thinspace | \thinspace \mathbf{w}_{mk}, \mathbf{X}_{nm})\right]^{c_{nk}}, \end{aligned}}  $$

where

$$p(\mathbf{y}_{nm} \thinspace | \thinspace \mathbf{z}_{nm}) = \mathbb{I}(\mathbf{z}_{nm} > 0)^{\mathbf{y}_{nm}} \mathbb{I}(\mathbf{z}_{nm} \leq 0)^{(\pmb{1} - \mathbf{y}_{nm})}. $$

To complete the model, we introduce priors over the parameters. We choose a Dirichlet distribution over the mixing proportions, $p(\boldsymbol {\pi }) = \mathcal {D}\text {ir}(\boldsymbol {\pi } \thinspace | \thinspace \boldsymbol {\delta }_{0})$, where for symmetry we choose the same parameter $\delta _{0_{k}}$ for each of the mixture components. We also introduce an independent Gaussian prior over the coefficients **W**, that is:

5 $$ p(\mathbf{W} \thinspace | \thinspace \boldsymbol{\tau}) = \prod_{m=1}^{M}\prod_{k=1}^{K} \mathcal{N}(\mathbf{w}_{mk} \thinspace | \thinspace \mathbf{0}, \tau_{k}^{-1} \mathbf{I}).  $$

**Fig. 7:**
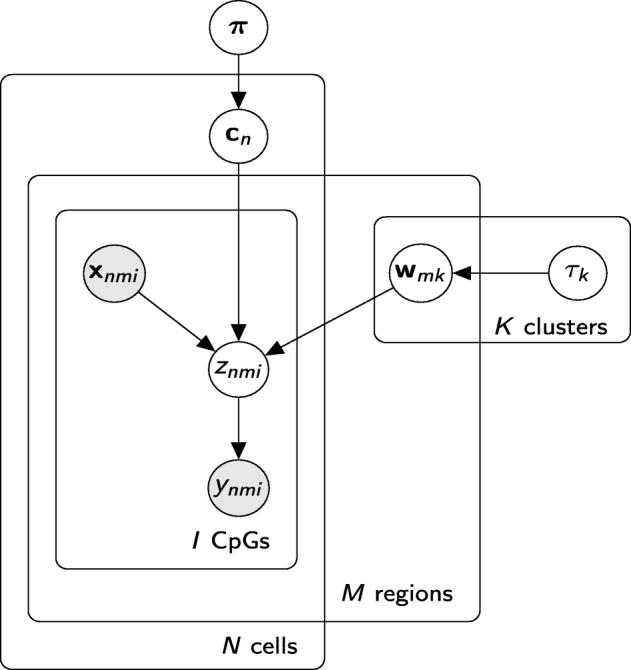
Probabilistic graphical representation of the Melissa model

Finally, we introduce a prior distribution for the (hyper)-parameter ***τ*** and assume that each cluster has its own precision parameter, $p(\tau _{k}) = \mathcal {G}\text {amma}(\tau _{k} \thinspace | \thinspace \alpha _{0}, \beta _{0})$. Having defined our model, we can now write the joint distribution over the observed and latent variables:

6 $$ {\begin{aligned} p(\mathbf{Y}, \mathbf{Z}, \mathbf{C}, \mathbf{W}, \boldsymbol{\pi}, \boldsymbol{\tau} \thinspace | \thinspace \mathbf{X}) = & p(\mathbf{Y} \thinspace | \thinspace \mathbf{Z}) \thinspace p(\mathbf{Z} \thinspace | \thinspace \mathbf{C}, \mathbf{W}, \mathbf{X}) \thinspace p(\mathbf{C} \thinspace | \thinspace \boldsymbol{\pi}) \thinspace p(\boldsymbol{\pi}) \thinspace p(\mathbf{W} \thinspace | \thinspace \boldsymbol{\tau}) \thinspace p(\boldsymbol{\tau}), \end{aligned}}  $$

where the factorization corresponds to the probabilistic graphical model shown in Fig. 7, resulting in the following hierarchical model:

$$ \begin{aligned} \boldsymbol{\pi} & \sim \mathcal{D}\text{ir}(\boldsymbol{\delta}_{0}) \\ \mathbf{c}_{n} \thinspace | \thinspace \boldsymbol{\pi} & \sim \mathcal{D}\text{iscrete}(\boldsymbol{\pi}) \\ \tau_{k} & \sim \mathcal{G}\text{amma}(\alpha_{0}, \beta_{0}) \\ \mathbf{w}_{mk} \thinspace | \thinspace \tau_{k} & \sim \mathcal{N}(\mathbf{0}, \tau_{k}^{-1}\mathbf{I}) \\ z_{nmi} \thinspace | \thinspace \mathbf{w}_{mk}, \mathbf{x}_{nmi} & \sim \mathcal{N}(\mathbf{w}_{mk}^{\top}\mathbf{x}_{nmi}, 1) \\ y_{nmi} \thinspace | \thinspace z_{nmi} & =\left\{ \begin{array}{ll} 1 & \; \text{if}~ z_{nmi} > 0\\ 0 & \; \text{if}~ z_{nmi} \leq 0.\\ \end{array} \right. \end{aligned} $$

Importantly, Melissa is a hybrid between a global unsupervised clustering model and a local supervised prediction model, encoded through the GLM regression coefficients **w** for each genomic region. When considering Melissa as an imputation (or predictive) model, the training data are coming by using only a subset of CpG tuples (*x*_*nmi*_,*y*_*nmi*_) for each region. For example, from the observed *I*_*nm*_ CpGs in a given region, Melissa will only see *I*_*nm*_/2 random CpGs during training, and the remaining CpGs will be used as a held out test set to evaluate its prediction performance. Note that in any case, either using all CpGs or a subset during training, Melissa will additionally perform clustering at the global level which is encoded through the latent variables **c**_*n*_.

#### Variational inference

The posterior distribution of the latent variables given the observed data *p*(**Z**,**C**,**W**,***π***,***τ*** | **Y**,**X**) for the Melissa model is not analytically tractable; hence, we resort to approximate techniques. The most common method for approximate Bayesian inference is to perform Markov Chain Monte Carlo (MCMC) [33]; however, sampling methods require considerable computational resources and do not scale well when performing genome-wide analysis on hundreds or thousands of single cells. Variational methods can provide an efficient, approximate solution with better scalability in this case (see the “Results” section for a comparison between Gibbs sampling and variational inference for this model). More specifically, we use mean-field variational inference [34] which assumes that the approximating distribution factorizes over the latent variables:

7 $$ q(\mathbf{Z}, \mathbf{C}, \mathbf{W}, \boldsymbol{\pi}, \boldsymbol{\tau}) = q(\mathbf{Z})\thinspace q(\mathbf{C}) \thinspace q(\mathbf{W}) \thinspace q(\boldsymbol{\pi}) \thinspace q(\boldsymbol{\tau}).  $$

Detailed mathematical derivations of the optimal variational factors are available in Additional file 1: Section 1. Next, we iteratively update each factor *q* while holding the remaining factors fixed using the coordinate ascent variational inference (CAVI) algorithm which is summarized in Algorithm 1.


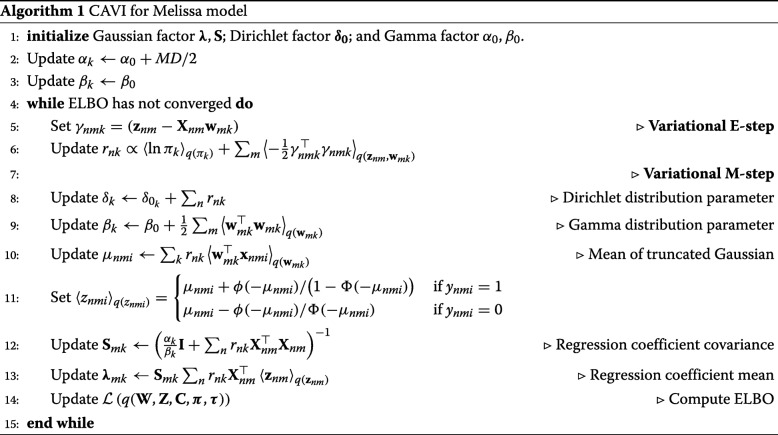


#### Predictive density and model selection

Given an approximate posterior distribution, we are in the position to predict the methylation level at unobserved CpG sites. The predictive density of a new observation **y**_∗_, which is associated with latent variables **c**_∗_, **z**_∗_ and covariates **X**_∗_, is given by:

8 $$ {\begin{aligned} p(\mathbf{y}_{*} \thinspace | \thinspace \mathbf{X}_{*},\mathbf{Y}) & = \sum_{c_{*}}\int\int p(\mathbf{y}_{*}, \mathbf{c}_{*}, \mathbf{z}_{*}, \boldsymbol{\theta} \thinspace | \thinspace \mathbf{X}_{*},\mathbf{Y}) d\boldsymbol{\theta}d\mathbf{z}_{*} \\ & \simeq \sum_{k=1}^{K}\frac{\delta_{k}}{\sum_{j}\delta_{j} }\mathcal{B}\text{ern}\left(\mathbf{y}_{*} \Big{|} \Phi \left(\frac{\mathbf{X}_{*}\boldsymbol{\lambda}_{k}}{\sqrt{\mathbf{I} + \text{diag}\big(\mathbf{X}_{*} \mathbf{S}_{k} \mathbf{X}_{*}^{T}\big)}}\right)\right) \end{aligned}}  $$

where we collectively denote as ***θ*** the relevant parameters being marginalized.

It has been repeatedly observed [26] that, when fitting variationally a mixture model with a large number of components, the variational procedure will prune away components with no support in the data, hence effectively determining an appropriate number of clusters in an automatic fashion, i.e., perform model selection. We can gain some intuition as to why this happens in the following way. We can rewrite the Kullback-Leibler ($\mathcal {KL}$) divergence as:

9 $$ {\begin{aligned} \mathcal{KL}(q(\boldsymbol{\theta})\;||\;p(\boldsymbol{\theta} \thinspace | \thinspace \mathbf{X})) = & \ln p(\mathbf{X}) - \left\langle \ln p(\mathbf{X} \thinspace | \thinspace \boldsymbol{\theta}) \right\rangle_{q(\boldsymbol{\theta})} + \mathcal{KL}(q(\boldsymbol{\theta})\;||\;p(\boldsymbol{\theta})) \end{aligned}}  $$

where ln*p*(**X**) can be ignored since it is constant with respect to *q*(***θ***). To minimize this objective function, the variational approximation will both try to increase the expected log likelihood of the data ln*p*(**X** | ***θ***) while minimizing its $\mathcal {KL}$ divergence with the prior distribution p (***θ***). Hence, using variational Bayes, we have an automatic trade-off between fitting the data and model complexity [19].

### Assessing Melissa via a simulation study

To generate realistic simulated single-cell methylation data, we first used the BPRMeth package [21] to infer five prototypical methylation profiles from the GM12878 lymphoblastoid cell line. The bulk BS-seq data for the GM12878 cell line are publicly available from the ENCODE project [35]. Based on these profiles, we simulated single-cell methylation data (i.e., binary CpG methylation states) for *M* = 100 genomic regions, where each CpG was generated by sampling from a Bernoulli distribution with probability of success given by the latent function evaluation at the specific site. To mimic the inherent noise introduced by bisulfite conversion error, Gaussian noise $\mathcal {N}(\mu = 0, \sigma = 0.05)$ was introduced to the probability of success prior to generating each binary CpG site. This process can be thought of as generating methylation data for a specific single cell. Next, we generated *K* = 4 cell sub-populations by randomly shuffling the genomic regions across clusters, so now each cell sub-population has its own methylome landscape. In total, we generated *N* = 200 cells, with the following cell sub-population proportions: 40%, 25%, 20%, and 15%. Additionally, to account for different levels of similarity between cell sub-populations, we simulated 11 different data sets by varying the proportion of similar genomic regions between clusters. Finally, to assess the performance of Melissa for varying number of cells assayed, we simulated 10 different data sets by varying the total number of single cells N. The scripts (written in the R programming language) for this simulation study are publicly available on the Melissa repository.

### Assessing Melissa on sub-sampled bulk ENCODE data

To faithfully simulate methylation data that resemble scBS-seq experiments, we generated two additional synthetic data sets by sub-sampling bulk ENCODE RRBS (GEO: GSE27584) and WGBS (GEO: GSE80911 for H1hESC and GSE86765 for GM12878) data, each consisting of two different cell lines, H1-hESC and GM12878. The RRBS data are enriched for genomic regions with high CpG content (using methylation sensitive restriction enzymes such as *MspI* that recognizes CCGG motifs) which predominantly reside near promoter regions and CpG islands. On the other hand, WGBS experiments in theory can assay the whole methylome landscape of the human genome; however, they require high-sequencing depth to obtain an accurate estimate of the bulk methylation level at each CpG site. To retain the structure of missing data observed in scBS-seq experiments (due to read length), we directly sub-sampled the raw FASTQ files which essentially lead to discarding individual reads rather than individual CpGs. For the RRBS data set, from each cell line, we generated 40 pseudo-single cells by randomly keeping 10% of the mapped reads from the bulk experiment, resulting in 80 cells when combining both cell lines. For the WGBS data set, the same number of pseudo-single cells was generated from each cell line, with the only difference that only 0.5% of the mapped reads were retained from the bulk data due to the high-sequencing depth of the experiments. This process was performed for chromosomes 1 to 6 to alleviate the computational burden. Subsequently, the same preprocessing steps detailed in the previous section were performed, with the only difference that for this study we considered only ± 2.5 kb and ± 5 kb promoter regions around TSS. Each model, except DeepCpG, used 20%, 50%, and 80% of the CpGs as training set, and the remaining of CpGs were used as a test set to evaluate imputation performance. The DeepCpG model used chromosomes 1 and 3 as training set, chromosome 5 as validation set, and the remaining chromosomes as test set.

### scBS-seq data and preprocessing

Single-cell bisulfite sequencing protocols provide us with single base-pair resolution of CpG methylation states. Since we assay the DNA of a single cell, the methylation level for each CpG site is predominantly binary, either methylated or unmethylated. However, due to each chromosome having two copies, a small proportion of CpG sites have a non-binary nature (see Additional file 1: Figure S19). To avoid ambiguities, hemi-methylated sites—sites with 50% methylation level—are filtered prior to downstream analysis, and for the remaining sites, binary methylation states are obtained from the ratio of methylated read counts to total read counts [11].

Two mouse embryonic stem cells (ESCs) data sets were used to validate the performance of the Melissa model. The scM&T-seq data set [11] after quality assessment consisted of 75 single cells out of which 14 cells were cultured in 2i medium (*2i ESCs*) and the remaining 61 cells were cultured in serum conditions (*serum ESCs*). The Bismark [36] processed data, with reads mapped to the GRCm38 mouse genome, were downloaded from the Gene Expression Omnibus under accession GSE74535. The scBS-seq data set [8] contained 32 cells out of which 12 cells were *2i ESCs* and the remaining 20 cells were *serum ESCs*, and the Bismark processed data, with reads mapped to the GRCm38 mouse genome, are publicly available under accession number GSE56879. For both data sets, the observed data that are used as input to Melissa are binary methylation states: unmethylated CpGs are encoded with zero and methylated CpGs with one. We should note that this is the standard procedure for processing scBS-seq data [8] and additional information and visualizations regarding the quality of the scBS-seq data can be found in the original publications.

Since Melissa considers genomic regions for a specific genomic context, we use the BPRMeth package [21] to filter CpGs that do not fall inside these regions, and create a simple data structure where each cell is a encoded as a list, and each entry of the list—corresponding to a specific genomic region—is a matrix with two columns: the (relative) CpG location and the methylation state. We considered six different genomic contexts where we applied Melissa: protein coding promoters with varying genomic windows: ± 1.5 kb, ± 2.5 kb, and ± 5 kb around transcription start sites (TSS), active enhancers, super enhancers, and Nanog regulatory regions. Due to the sparse CpG coverage, for the three genomic contexts except promoters, we filtered loci with smaller than 1 kb annotation length, and specifically for Nanog regions, we took a window of ± 2.5 kb around the center of the genomic annotation. In addition, we only considered regions that were covered in at least 50% of the cells with a minimum coverage of 10 CpGs and had between cell variability; the rationale being that homogeneous regions across cells do not provide additional information for identifying cell sub-populations. The CpG coverage distribution after the filtering process across different genomic contexts is shown in Additional file 1: Figure S20 and S21. The sparsity level of the two scBS-seq data sets across different genomic contexts is shown in Additional file 1: Table S3. It should be noted that imputation performance is evaluated only on genomic regions that pass the filtering threshold. We run the model with *K*=6 and *K*=5 clusters for the scM&T-seq and scBS-seq data sets, respectively, and we use a broad prior over the model parameters.

### Performance evaluation

To assess model performance across all genomic contexts, we partition the data and use 50% of the CpGs in each cell and region for training set and the remaining 50% as test set (except DeepCpG, see below). The prediction performance of all competing models, except DeepCpG, was evaluated on imputing all missing CpG states in a given region at once. For computing binary evaluation metrics, such as *F*-measure, predicted probabilities above 0.5 were set to one and rounded to zero otherwise.

***F***-*measure* The *F*-measure or *F*_1_-score is the harmonic mean of precision and recall:

10 $$ F\text{-measure} = 2 \cdot \frac{\text{precision} \cdot \text{recall}}{\text{precision} + \text{recall}}.  $$

**Gaussian mixture model** The input to the Gaussian mixture model (GMM) is the average methylation rate across the region; since rates are between (0,1), we transform them to *M* values, which follow closer the Gaussian distribution [22]. The transformation from average methylation rates to average *M* values is obtained by:

11 $$ M\text{value} = \log_{2}\left(\frac{\text{rate} + 0.01}{1 - \text{rate} + 0.01}\right).  $$

**Adjusted Rand Index** The Adjusted Rand Index (ARI) is a measure of the similarity between two data clusterings:

12 $$ ARI = \frac{\sum_{ij} \binom{n_{ij}}{2} - \left[\sum_{i} \binom{\alpha_{i}}{2}\sum_{j}\binom{\beta_{j}}{2} \right] / \binom{n}{2}}{\frac{1}{2} \left[ \sum_{i} \binom{\alpha_{i}}{2} + \sum_{j}\binom{\beta_{j}}{2}\right] - \left[\sum_{i} \binom{\alpha_{i}}{2}\sum_{j}\binom{\beta_{j}}{2} \right] / \binom{n}{2}}.  $$

#### DeepCpG

The DeepCpG method takes a different imputation approach: it is trained on a specific set of chromosomes and predicts methylation states on the remaining chromosomes where it imputes each CpG site sequentially by using as input a set of neighboring CpG sites. This approach makes it difficult to equally compare with the rival methods, since for each CpG the input features to DeepCpG are all the neighboring sites, whereas the competing models have access to a subset of the data and they make predictions in one pass for the whole region. Since we only had access to CpG methylation data and to make it comparable with the considered methods, we trained the CpG module of DeepCpG (termed *DeepCpG CpG* in [24]).

For the scM&T-seq data set, chromosomes 3 and 17 were used as training set, chromosomes 12 and 14 as validation set and the remaining chromosomes as test set. For the scBS-seq data set, chromosomes 3, 17, and 19 were used as training set; chromosomes 12 and 14 as validation set; and the remaining chromosomes as test set. The chosen chromosomes had at least three million CpGs used as training set, a sensible size for the DeepCpG model as suggested by the authors. A neighborhood of *K* = 20 CpG sites to the left and the right for each target CpG was used as input to the model. During testing time, even if a given genomic region did not contain at least 40 CpGs, the DeepCpG model used additional CpGs outside this window to predict methylation states, hence using more information compared to the rival models. In total, the DeepCpG model took around 4 days per data set for training and prediction on a cluster equipped with NVIDIA Tesla K40ms GPUs.

## Additional file

Additional file 1 Melissa mean-field variational inference derivations (section 1), additional figures (section 2), and additional tables (Section 3). (PDF 884 kb)

## Abbreviations

ARI: Adjusted Rand Index
AUC: Area under the receiver operating characteristic curve
BPRMeth: Bayesian probit regression for methylation
bp: Base pair
CAVI: Coordinate ascent variational inference
CPU: Central processing unit
ESC: Embryonic stem cell
GEO: Gene expression omnibus
GLM: Generalized linear model
GMM: Gaussian mixture model
GPU: Graphics processing unit
MCMC: Markov chain Monte Carlo
RBF: Radial basis function
RF: Random Forest
scBS-seq: Single-cell bisulfite sequencing
scRRBS: Single-cell reduced representation bisulfite sequencing
TSS: Transcription start site
WGBS: Whole genome bisulfite sequencing
