# Supplementary material for: Melissa: Bayesian clustering and imputation of single-cell methylomes
Source: Genome Biol. 2019 Mar 21;20:61. doi: 10.1186/s13059-019-1665-8 (PMC6427844; doi:10.1186/s13059-019-1665-8)
Supplement: Supplementary file 1 — Melissa mean-field variational inference derivations (section 1), additional figures (section 2), and additional tables (Section 3). (PDF 884 kb) [file 13059_2019_1665_MOESM1_ESM.pdf]

Additional information for the paper

Melissa: Bayesian clustering and imputation of single cell methylomes

Chantriolnt-Andreas Kapourani[1,2,*] C.A.Kapourani@ed.ac.uk
Guido Sanguinetti[1,3,*] G.Sanguinetti@ed.ac.uk

[1]School of Informatics, University of Edinburgh, Edinburgh EH8 9AB, UK
[2]MRC Institute of Genetics and Molecular Medicine, University of Edinburgh, Edinburgh EH4 2XU, UK
[3]Synthetic and Systems Biology, University of Edinburgh, Edinburgh EH9 3BF, UK
[*]To whom correspondence should be addressed

1 Melissa: mean-field variational inference derivations

In mean-field variational inference the intractable posterior distribution of the latent variables $p(\boldsymbol{\theta} \,|\, \mathbf{X})$ is approximated by a factorized distribution $q(\boldsymbol{\theta}) = \prod_i q_i(\boldsymbol{\theta}_i)$, where $\boldsymbol{\theta}$ denotes the latent variables and $\mathbf{X}$ the observed variables. Then we search over the space of approximating distributions to find the distribution with the minimum Kullback-Leibler ($\mathcal{KL}$) divergence with the actual posterior

$$\mathcal{KL}(q(\boldsymbol{\theta}) \,||\, p(\boldsymbol{\theta} \,|\, \mathbf{X})) = -\int q(\boldsymbol{\theta}) \ln \frac{p(\boldsymbol{\theta} \,|\, \mathbf{X})}{q(\boldsymbol{\theta})} d\boldsymbol{\theta}. \tag{1}$$

The $\mathcal{KL}$ divergence can then be minimised by performing a free form minimisation over the $q_i(\boldsymbol{\theta}_i)$ leading to the following update equation

$$q_i(\boldsymbol{\theta}_i) = \frac{\exp \left\langle \ln p(\mathbf{X}, \boldsymbol{\theta}) \right\rangle_{q_{j \neq i}}}{\int \exp \left\langle \ln p(\mathbf{X}, \boldsymbol{\theta}) \right\rangle_{q_{j \neq i}} d\boldsymbol{\theta}_i}, \tag{2}$$

where $\langle \cdot \rangle_{q_{j \neq i}}$ denotes an expectation with respect to the distributions $q_j(\boldsymbol{\theta}_j)$ for all $j \neq i$. The joint distribution over the observed and latent variables for the Melissa model is

$$p(\mathbf{Y}, \mathbf{Z}, \mathbf{C}, \mathbf{W}, \boldsymbol{\pi}, \boldsymbol{\tau} | \mathbf{X}) = p(\mathbf{Y}|\mathbf{Z})\; p(\mathbf{Z}|\mathbf{C}, \mathbf{W}, \mathbf{X})\; p(\mathbf{C}|\boldsymbol{\pi}) p(\boldsymbol{\pi})\; p(\mathbf{W}|\boldsymbol{\tau})\; p(\boldsymbol{\tau}), \tag{3}$$

where the factorisation corresponds to the probabilistic graphical model shown in Figure 7 in the main text. We assume that the variational approximation to our posterior distribution factorises over the latent variables (mean-field variational inference)

$$q(\mathbf{Z}, \mathbf{C}, \mathbf{W}, \boldsymbol{\pi}, \boldsymbol{\tau}) = q(\mathbf{Z})\; q(\mathbf{C})\; q(\mathbf{W})\; q(\boldsymbol{\pi})\; q(\boldsymbol{\tau}). \tag{4}$$

1.1 Deriving optimised factors

Below we derive the optimised factors of the variational posterior using Equation (2).

Factor $q(\mathbf{C})$

$$\begin{aligned}
\ln q(\mathbf{C}) &= \langle \ln p(\mathbf{Y}, \mathbf{Z}, \mathbf{C}, \boldsymbol{\pi}, \mathbf{W}, \boldsymbol{\tau} | \mathbf{X}) \rangle_{q(\mathbf{Z},\boldsymbol{\pi},\mathbf{W},\boldsymbol{\tau})} + \text{const} \\
&= \left\langle \ln \left\{ \underbrace{p(\mathbf{Y}|\mathbf{Z})}_{\text{const}} \; p(\mathbf{Z}|\mathbf{C}, \mathbf{W}, \mathbf{X}) \; p(\mathbf{C}|\boldsymbol{\pi}) \; \underbrace{p(\boldsymbol{\pi}) \; p(\mathbf{W}|\boldsymbol{\tau}) \; p(\boldsymbol{\tau})}_{\text{const}} \right\} \right\rangle_{q(\mathbf{Z},\boldsymbol{\pi},\mathbf{W},\boldsymbol{\tau})} + \text{const} \\
&= \sum_{n=1}^{N} \sum_{k=1}^{K} c_{nk} \sum_{m=1}^{M} \langle \ln \mathcal{N}(\mathbf{z}_{nm} | \mathbf{X}_{nm} \mathbf{w}_{mk}, \mathbf{I}_{nm}) \rangle_{q(\mathbf{z_{nm}}, \mathbf{w}_{mk})} + \sum_{n=1}^{N} \sum_{k=1}^{K} c_{nk} \; \langle \ln \pi_k \rangle_{q(\pi_k)} + \text{const} \\
&= \sum_{n=1}^{N} \sum_{k=1}^{K} c_{nk} \; \ln \rho_{nk} + \text{const},
\end{aligned} \tag{5}$$

where

$$\ln \rho_{nk} = \sum_{m=1}^{M} \left\langle -\frac{1}{2} \Big(\mathbf{z}_{nm} - \mathbf{X}_{nm} \mathbf{w}_{mk} \Big)^T \Big(\mathbf{z}_{nm} - \mathbf{X}_{nm} \mathbf{w}_{mk} \Big) \right\rangle_{q(\mathbf{z}_{nm}, \mathbf{w}_{mk})} + \langle \ln \pi_k \rangle_{q(\pi_k)} .$$

Taking the exponential on both sides and requiring that this distribution be normalised we obtain

$$q(\mathbf{C}) = \prod_{n=1}^{N} \prod_{k=1}^{K} r_{nk}^{c_{nk}} \qquad \text{where} \quad r_{nk} = \frac{\rho_{nk}}{\sum_{j=1}^{K} \rho_{nj}} .$$

Factor $q(\boldsymbol{\tau})$

$$\begin{aligned}
\ln q(\boldsymbol{\tau}) &= \left\langle \ln \left\{ \underbrace{p(\mathbf{Y}|\mathbf{Z}) \; p(\mathbf{Z}|\mathbf{C}, \mathbf{W}, \mathbf{X}) \; p(\mathbf{C}|\boldsymbol{\pi}) \; p(\boldsymbol{\pi})}_{\text{const}} \; p(\mathbf{W}|\boldsymbol{\tau}) \; p(\boldsymbol{\tau}) \right\} \right\rangle_{q(\mathbf{Z},\mathbf{C},\boldsymbol{\pi},\mathbf{W})} + \text{const} \\
&= \langle \ln p(\mathbf{W}|\boldsymbol{\tau}) \rangle_{q(\mathbf{W})} + \ln p(\boldsymbol{\tau}) + \text{const} \\
&= \sum_{k=1}^{K} \sum_{m=1}^{M} \langle \ln p(\mathbf{w}_{mk} | \tau_k) \rangle_{q(\mathbf{w}_{mk})} + \sum_{k=1}^{K} \ln p(\tau_k) + \text{const}.
\end{aligned} \tag{6}$$

Here we observe that the right hand side comprises a sum over k, i.e. each τ_k is independent of each other, hence

$$\begin{aligned}
\ln q(\tau_k) &= \sum_{m=1}^{M} \langle \ln p(\mathbf{w}_{mk} | \tau_k) \rangle_{q(\mathbf{w}_{mk})} + \ln p(\tau_k) + \text{const} \\
&= \underbrace{\frac{MD}{2} \ln \tau_k - \frac{\tau_k}{2} \sum_{m=1}^{M} \left\langle \mathbf{w}_{mk}^T \mathbf{w}_{mk} \right\rangle_{q(\mathbf{w}_{mk})}}_{\text{Gaussian PDF}} + \underbrace{(\alpha_0 - 1) \ln \tau_k - \beta_0 \tau_k}_{\text{Gamma PDF}} \\
&= \underbrace{(\alpha_0 + \frac{MD}{2} - 1)}_{\alpha_k \text{ parameter}} \ln \tau_k - \Big(\underbrace{\beta_0 + \frac{1}{2} \sum_{m=1}^{M} \left\langle \mathbf{w}_{mk}^T \mathbf{w}_{mk} \right\rangle_{q(\mathbf{w}_{mk})}}_{\beta_k \text{ parameter}} \Big) \tau_k,
\end{aligned} \tag{7}$$

which is the logarithm of the (un-normalised) Gamma distribution, leading to

$$\begin{aligned}
q(\tau_k) &= \mathcal{G}\text{amma}(\tau_k | \alpha_k, \beta_k) \\
\alpha_k &= \alpha_0 + \frac{MD}{2} \\
\beta_k &= \beta_0 + \frac{1}{2} \sum_{m=1}^{M} \left\langle \mathbf{w}_{mk}^T \mathbf{w}_{mk} \right\rangle_{q(\mathbf{w}_{mk})} .
\end{aligned} \tag{8}$$

Note that the update for the α hyperparameter depends only on the total number of genomic regions and the number of basis functions used to estimate the underlying methylation profiles. On the other hand the β hyperparameter is updated at each CAVI iteration, since it depends on the expected value of the regression coefficients. The expected value of the $\mathcal{G}$amma distribution is $E = \alpha/\beta$, and the inverse of this quantity is the variance parameter for the prior Gaussian distribution of the coefficients $\mathbf{w}$. Large values of E result in small variance Gaussian priors, hence the model is substantially penalised when weights are moving away from prior mean $\mu_0 = 0$; as a consequence the model will tend to prune away clusters, that is, set all weights $\mathbf{w}_{mk} = \mathbf{0}$. This may strongly affect the model in the initial iterations of CAVI, which will affect the β parameter but not the α parameter of the $\mathcal{G}$amma distribution, potentially leading to convergence to a suboptimal local maximum. Hence, one should be cautious when setting the initial values for these parameters; in the current implementation of Melissa we set $\alpha_0 = 0.5$ and $\beta_0 = \sqrt{a_k}$.

Factor $q(\boldsymbol{\pi})$

$$
\begin{aligned}
\ln q(\boldsymbol{\pi}) &= \left\langle \ln \left\{ \underbrace{p(\mathbf{Y}|\mathbf{Z})\, p(\mathbf{Z}|\mathbf{C},\mathbf{W},\mathbf{X})}_{\text{const}}\; p(\mathbf{C}|\boldsymbol{\pi})\, p(\boldsymbol{\pi})\; \underbrace{p(\mathbf{W}|\boldsymbol{\tau})\, p(\boldsymbol{\tau})}_{\text{const}} \right\} \right\rangle_{q(\mathbf{Z},\mathbf{W},\mathbf{C},\boldsymbol{\tau})} + \text{const} \\
&= \ln p(\boldsymbol{\pi}) + \langle \ln p(\mathbf{C}|\boldsymbol{\pi}) \rangle_{q(\mathbf{C})} + \text{const} \\
&= \underbrace{\ln C(\boldsymbol{\delta}_0)}_{\text{const}} + \sum_{k=1}^{K} \ln \pi_k^{\delta_{0_k}-1} + \sum_{k=1}^{K}\sum_{n=1}^{N} \underbrace{\langle c_{nk} \rangle_{q(c_{nk})}}_{r_{nk}} \ln \pi_k + \text{const} \\
&= \sum_{k=1}^{K} \ln \pi_k^{\delta_{0_k}-1} + \sum_{k=1}^{K}\sum_{n=1}^{N} r_{nk} \ln \pi_k + \text{const.}
\end{aligned} \tag{9}
$$

Taking the exponential on both sides we observe that $q(\boldsymbol{\pi})$ is a Dirichlet distribution

$$
\begin{aligned}
q(\boldsymbol{\pi}) &= \prod_{k=1}^{K} \pi_k^{(\delta_{0_k} + \sum_{n=1}^{N} r_{nk} - 1)} \\
&= \mathcal{D}ir(\boldsymbol{\pi}|\boldsymbol{\delta}).
\end{aligned} \tag{10}
$$

where $\boldsymbol{\delta}$ has components δ_k given by $\delta_k = \delta_{0_k} + \sum_{n=1}^{N} r_{nk}$.

Factor $q(\mathbf{W})$

$$
\begin{aligned}
\ln q(\mathbf{w}_{mk}) &= \left\langle \ln \left\{ \underbrace{p(\mathbf{Y}|\mathbf{Z})}_{\text{const}}\; p(\mathbf{Z}|\mathbf{C},\mathbf{W},\mathbf{X})\; \underbrace{(\mathbf{C}|\boldsymbol{\pi})\, p(\boldsymbol{\pi})}_{\text{const}}\; p(\mathbf{W}|\boldsymbol{\tau})\; \underbrace{p(\boldsymbol{\tau})}_{\text{const}} \right\} \right\rangle_{q(\mathbf{Z},\mathbf{C},\boldsymbol{\pi},\boldsymbol{\tau})} + \text{const} \\
&= \sum_{n=1}^{N} \langle c_{nk} \rangle_{q(c_{nk})} \; \langle \ln \mathcal{N}(\mathbf{z}_{nm}|\mathbf{X}_{nm}\mathbf{w}_{mk}, \mathbf{I}_{nm}) \rangle_{q(\mathbf{z}_{nm})} + \langle \ln p(\mathbf{w}_{mk}|\tau_k) \rangle_{q(\tau_k)} + \text{const} \\
&= \sum_{n=1}^{N} r_{nk} \left\langle -\frac{1}{2}\Big(\mathbf{z}_{nm} - \mathbf{X}_{nm}\mathbf{w}_{mk}\Big)^T \Big(\mathbf{z}_{nm} - \mathbf{X}_{nm}\mathbf{w}_{mk}\Big) \right\rangle_{q(\mathbf{z}_{nm})} - \frac{1}{2} \langle \tau_k \rangle_{q(\tau_k)} \mathbf{w}_{mk}^T \mathbf{w}_{mk} + \text{const} \\
&= \sum_{n=1}^{N} r_{nk} \left\{ \mathbf{w}_{mk}^T \mathbf{X}_{nm}^T \; \langle \mathbf{z}_{nm} \rangle_{q(\mathbf{z}_{nm})} - \frac{1}{2} \mathbf{w}_{mk}^T \mathbf{X}_{nm}^T \mathbf{X}_{nm} \mathbf{w}_{mk} \right\} - \frac{1}{2} \langle \tau_k \rangle_{q(\tau_k)} \mathbf{w}_{mk}^T \mathbf{w}_{mk} + \text{const} \\
&= \mathbf{w}_{mk}^T \sum_{n=1}^{N} r_{nk} \mathbf{X}_{nm}^T \; \langle \mathbf{z}_{nm} \rangle_{q(\mathbf{z}_{nm})} - \frac{1}{2} \mathbf{w}_{mk}^T \left\{ \langle \tau_k \rangle_{q(\tau_k)} \mathbf{I} + \sum_{n=1}^{N} r_{nk} \mathbf{X}_{nm}^T \mathbf{X}_{nm} \right\} \mathbf{w}_{mk} + \text{const.}
\end{aligned} \tag{11}
$$

Because this is a quadratic form, the distribution $q(\mathbf{w}_{mk})$ is a Gaussian distribution and we can complete the square to identify the mean and the covariance matrix

$$
\begin{aligned}
q(\mathbf{w}_{mk}) &= \mathcal{N}(\mathbf{w}_{mk}|\boldsymbol{\lambda}_{mk}, \mathbf{S}_{mk}) \\
\boldsymbol{\lambda}_{mk} &= \mathbf{S}_{mk} \sum_{n=1}^{N} r_{nk} \mathbf{X}_{nm}^T \ \langle \mathbf{z}_{nm} \rangle_{q(\mathbf{z}_{nm})} \\
\mathbf{S}_{mk} &= \left(\langle \tau_k \rangle_{q(\tau_k)} \mathbf{I} + \sum_{n=1}^{N} r_{nk} \mathbf{X}_{nm}^T \mathbf{X}_{nm} \right)^{-1} .
\end{aligned} \tag{12}
$$

Factor $q(\mathbf{Z})$ The log of the optimised factor assuming that the corresponding $y_{nmi} = 1$ is

$$
\begin{aligned}
\ln q(z_{nmi}) &= \left\langle \ln \left\{ p(\mathbf{Y}|\mathbf{Z})\ p(\mathbf{Z}|\mathbf{C}, \mathbf{W}, \mathbf{X})\ \underbrace{p(\mathbf{C}|\boldsymbol{\pi})\ p(\boldsymbol{\pi})\ p(\mathbf{W}|\boldsymbol{\tau})\ p(\boldsymbol{\tau})}_{\text{const}} \right\} \right\rangle_{q(\mathbf{C}, \boldsymbol{\pi}, \mathbf{W}, \boldsymbol{\tau})} + \text{const} \\
&= \ln p(y_{nmi}|z_{nmi}) + \left\langle \ln \prod_{k=1}^{K} \mathcal{N}(z_{nmi}|\mathbf{w}_{mk}^T \mathbf{x}_{nmi}, 1)^{c_{nk}} \right\rangle_{q(\mathbf{c}_n, \mathbf{w}_m)} + \text{const} \\
&= y_{nmi} \ln \mathbf{1}(z_{nmi} > 0) + \underbrace{(1 - y_{nmi}) \ln \mathbf{1}(z_{nmi} \leq 0)}_{0, \text{ since } y_{nmi}=1} + \\
&\qquad \sum_{k=1}^{K} r_{nk} \ \langle \ln \mathcal{N}(z_{nmi}|\mathbf{w}_{mk}^T \mathbf{x}_{nmi}, 1) \rangle_{q(\mathbf{w}_{mk})} + \text{const} \\
&= \ln \mathbf{1}(z_{nmi} > 0) - \frac{1}{2} z_{nmi}^2 \underbrace{\sum_{k=1}^{K} r_{nk}}_{1} + z_{nmi} \sum_{k=1}^{K} r_{nk} \ \langle \mathbf{w}_{mk}^T \rangle_{q(\mathbf{w}_{mk})} \mathbf{x}_{nmi} + \text{const}.
\end{aligned} \tag{13}
$$

Exponentiating this quantity and setting $\mu_{nmi} = \sum_{k=1}^{K} r_{nk} \langle \mathbf{w}_{mk}^T \rangle_{q(\mathbf{w}_{mk})} \mathbf{x}_{nmi}$ we obtain

$$
q(z_{nmi}) \propto \mathbf{1}(z_{nmi} > 0) \exp \left(-\frac{1}{2} z_{nmi}^2 + z_{nmi} \mu_{nmi} \right) . \tag{14}
$$

We observe that the optimized factor $q(z_{nmi})$ is an un-normalised Truncated Normal distribution

$$
q(z_{nmi}) = \begin{cases} \mathcal{TN}_{+} (z_{nmi}|\mu_{nmi}, 1) & \text{if } y_{nmi} = 1 \\ \mathcal{TN}_{-} (z_{nmi}|\mu_{nmi}, 1) & \text{if } y_{nmi} = 0. \end{cases} \tag{15}
$$

1.2 Variational lower bound

The variational lower bound $\mathcal{L}(q)$ (i.e. evidence lower bound (ELBO)) is given by

$$
\begin{aligned}
\mathcal{L}(q) &= \sum_{\mathbf{C}} \int \int \int \int q(\mathbf{Z}, \mathbf{C}, \boldsymbol{\pi}, \mathbf{W}, \boldsymbol{\tau}) \ln \left\{ \frac{p(\mathbf{Y}, \mathbf{Z}, \mathbf{C}, \boldsymbol{\pi}, \mathbf{W}, \boldsymbol{\tau}|\mathbf{X})}{q(\mathbf{Z}, \mathbf{C}, \boldsymbol{\pi}, \mathbf{W}, \boldsymbol{\tau})} \right\} d\mathbf{Z}\, d\boldsymbol{\pi}\, d\mathbf{W}\, d\boldsymbol{\tau} \\
&= \langle \ln p(\mathbf{Y}, \mathbf{Z}, \mathbf{C}, \boldsymbol{\pi}, \mathbf{W}, \boldsymbol{\tau}|\mathbf{X}) \rangle_{q(\mathbf{Z}, \mathbf{C}, \boldsymbol{\pi}, \mathbf{W}, \boldsymbol{\tau})} - \langle \ln q(\mathbf{Z}, \mathbf{C}, \boldsymbol{\pi}, \mathbf{W}, \boldsymbol{\tau}) \rangle_{q(\mathbf{Z}, \mathbf{C}, \boldsymbol{\pi}, \mathbf{W}, \boldsymbol{\tau})} \\
&= \langle \ln p(\mathbf{Y}|\mathbf{Z}) \rangle_{q(\mathbf{Z})} + \langle \ln p(\mathbf{Z}|\mathbf{C}, \mathbf{W}, \mathbf{X}) \rangle_{q(\mathbf{Z}, \mathbf{C}, \mathbf{W})} + \langle \ln p(\mathbf{C}|\boldsymbol{\pi}) \rangle_{q(\mathbf{C}, \boldsymbol{\pi})} + \langle \ln p(\boldsymbol{\pi}) \rangle_{q(\boldsymbol{\pi})} \\
&\quad + \langle \ln p(\mathbf{W}|\boldsymbol{\tau}) \rangle_{q(\mathbf{W}, \boldsymbol{\tau})} + \langle \ln p(\boldsymbol{\tau}) \rangle_{q(\boldsymbol{\tau})} - \langle \ln q(\mathbf{Z}) \rangle_{q(\mathbf{Z})} - \langle \ln q(\mathbf{C}) \rangle_{q(\mathbf{C})} \\
&\quad - \langle \ln q(\boldsymbol{\pi}) \rangle_{q(\boldsymbol{\pi})} - \langle \ln q(\mathbf{W}) \rangle_{q(\mathbf{W})} - \langle \ln q(\boldsymbol{\tau}) \rangle_{q(\boldsymbol{\tau})} .
\end{aligned} \tag{16}
$$

We can derive the expectations in a similar fashion to Section 1.1. The ELBO $\mathcal{L}(q)$ is used to assess convergence of the coordinate ascent variational inference (CAVI) algorithm (Blei *et al.*, 2017).

1.3 Predictive density

The predictive density of a new observation $\mathbf{y}_*$ which will be associated with a latent variable $\mathbf{c}_*$, latent observation $\mathbf{z}_*$ and covariates $\mathbf{X}_*$ is given by

$$
\begin{aligned}
p(\mathbf{y}_*|\mathbf{X}_*, \mathbf{Y}, \mathbf{X}) &= \sum_c \int p(\mathbf{y}_*, \mathbf{c}_*, \mathbf{z}_*, \boldsymbol{\pi}, \mathbf{W}, \boldsymbol{\tau}|\mathbf{X}_*, \mathbf{Y}, \mathbf{X})\; d\boldsymbol{\pi}\; d\boldsymbol{\tau}\; d\mathbf{W}\; d\mathbf{z}_* \\
&= \sum_c \int p(\mathbf{y}_*|\mathbf{z}_*) p(\mathbf{z}_*|\mathbf{c}_*, \mathbf{W}, \mathbf{X}_*) p(\mathbf{c}_*|\boldsymbol{\pi}) p(\boldsymbol{\pi}, \mathbf{W}, \boldsymbol{\tau}|\mathbf{Y}, \mathbf{X})\; d\boldsymbol{\pi}\; d\boldsymbol{\tau}\; d\mathbf{W}\; d\mathbf{z}_* \\
&\simeq \sum_{k=1}^{K} \int p(\mathbf{y}_*|\mathbf{z}_*) p(\mathbf{z}_*|\mathbf{W}_k, \mathbf{X}_*) \pi_k\; q(\boldsymbol{\pi}) q(\mathbf{W}_k) q(\tau_k)\; d\boldsymbol{\pi}\; d\tau_k\; d\mathbf{W}_k\; d\mathbf{z}_* \\
&= \sum_{k=1}^{K} \frac{\delta_k}{\hat{\delta}} \int p(\mathbf{y}_*|\mathbf{z}_*) \mathcal{N}(\mathbf{z}_*|\mathbf{X}_*\mathbf{W}_k, \mathbf{I}_n) \mathcal{N}(\mathbf{W}_k|\boldsymbol{\lambda}_k, \mathbf{S}_k)\; d\mathbf{W}_k\; d\mathbf{z}_* \\
&= \sum_{k=1}^{K} \frac{\delta_k}{\hat{\delta}} \int p(\mathbf{y}_*|\mathbf{z}_*) \mathcal{N}\left(\mathbf{z}_*|\mathbf{X}_*\boldsymbol{\lambda}_k,\; \mathbf{I}_n + \operatorname{diag}\big(\mathbf{X}_*\mathbf{S}_k\mathbf{X}_*^T\big)\right)\; d\mathbf{z}_* \\
&= \sum_{k=1}^{K} \frac{\delta_k}{\hat{\delta}} \begin{cases} \int_0^\infty \mathcal{N}\left(\mathbf{z}_*|\mathbf{X}_*\boldsymbol{\lambda}_k,\; \mathbf{I}_n + \operatorname{diag}\big(\mathbf{X}_*\mathbf{S}_k\mathbf{X}_*^T\big)\right)\; dz & \text{where } \mathbf{y}_* = 1 \\ \int_{-\infty}^0 \mathcal{N}\left(\mathbf{z}_*|\mathbf{X}_*\boldsymbol{\lambda}_k,\; \mathbf{I}_n + \operatorname{diag}\big(\mathbf{X}_*\mathbf{S}_k\mathbf{X}_*^T\big)\right)\; dz & \text{where } \mathbf{y}_* = 0 \end{cases} \\
&= \sum_{k=1}^{K} \frac{\delta_k}{\hat{\delta}} \Phi\left(\rho\right)^{\mathbf{y}_*} \left(1 - \Phi\left(\rho\right)\right)^{(1-\mathbf{y}_*)} \\
&= \sum_{k=1}^{K} \frac{\delta_k}{\hat{\delta}} \mathcal{B}\mathrm{ern}\left(\mathbf{y}_* \middle| \Phi\left(\rho\right)\right).
\end{aligned} \tag{17}
$$

where $\hat{\delta} = \sum_k \delta_k$, $\Phi(\cdot)$ denotes the cumulative distribution function (cdf) of the standard normal distribution and

$$
\rho = \frac{\mathbf{X}_*\boldsymbol{\lambda}_k}{\big(\mathbf{I}_n + \operatorname{diag}\big(\mathbf{X}_*\mathbf{S}_k\mathbf{X}_*^T\big)\big)^{1/2}}. \tag{18}
$$

2 Additional figures

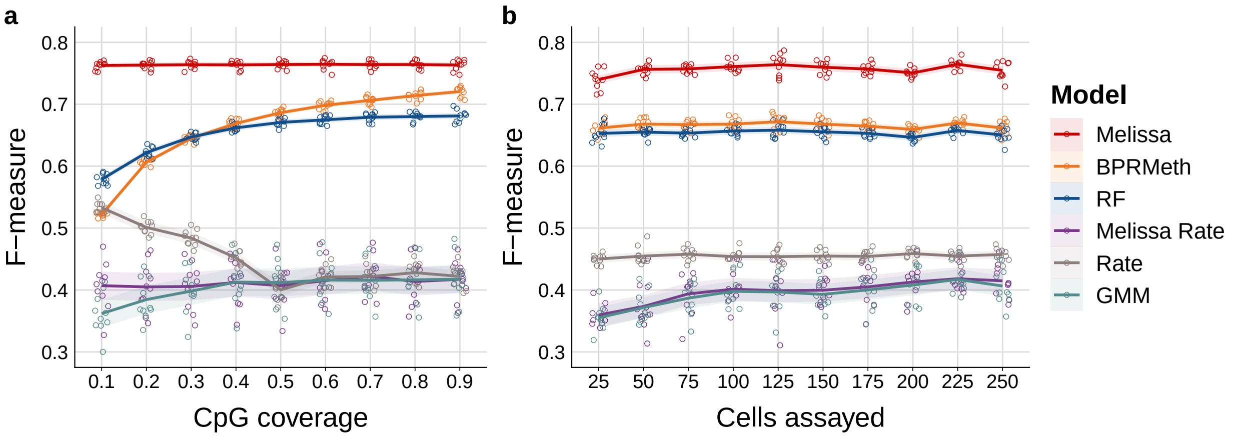

Figure S1: Melissa robustly imputes CpG methylation states. (**a**) Imputation performance in terms of F-measure as we vary the proportion of covered CpGs used for training. Higher values correspond to better imputation performance. For each CpG coverage setting a total of 10 random splits of the data to training and test sets was performed. Each coloured circle corresponds to a different simulation. The plot shows also the LOESS curve for each method as we increase CpG coverage. (**b**) Imputation performance measured by F-measure for varying number of cells assayed. In (**a**) N = 200 cells were simulated and cluster dissimilarity was set to 0.5, and in (**b**) CpG coverage was set to 0.4 and cluster dissimilarity to 0.5.

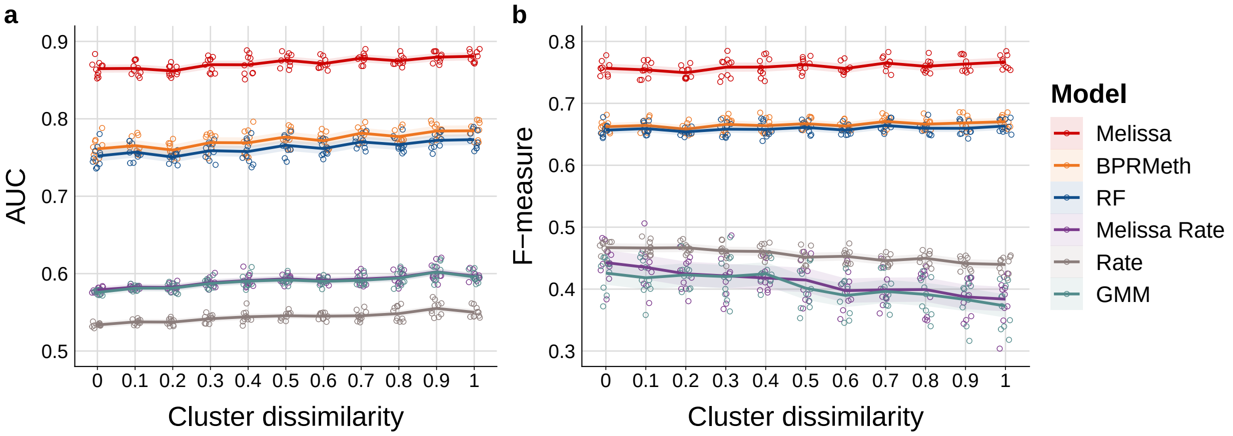

Figure S2: Melissa robustly imputes CpG methylation states for different levels of dissimilarity across clusters; values closer to zero correspond to highly similar cell sub-populations, whereas values closer to one correspond to well separated cell sub-populations. Imputation performance is measured in terms of (**a**) AUC and (**b**) F-measure. Higher values correspond to better imputation performance. For each CpG coverage setting a total of 10 random splits of the data to training and test sets was performed. Each coloured circle corresponds to a different simulation. The plot shows also the LOESS curve for each method as we increase the cluster dissimilarity across cell sub-populations. The CpG coverage was set to 0.4 and a total of N = 200 cells were simulated per experiment.

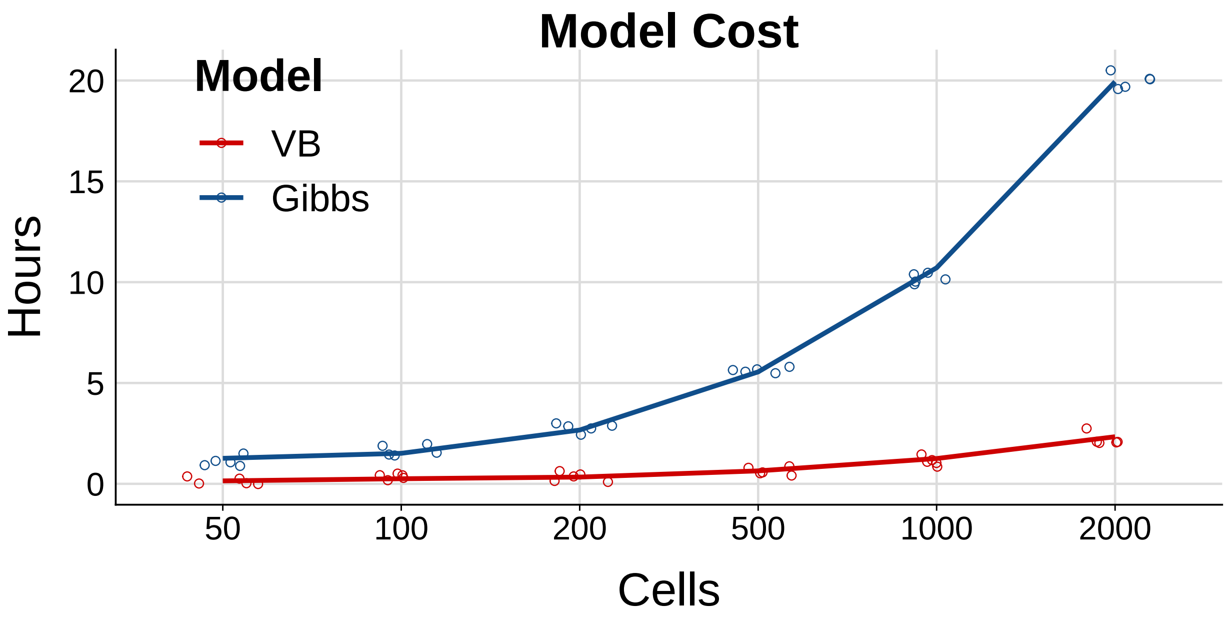

Figure S3: Melissa efficiently imputes and clusters cell sub-populations. Running times for varying number of cells for the variational Bayes (VB) and Gibbs sampling implementations for the Melissa model, where each cell consists of $M = 200$ genomic regions.

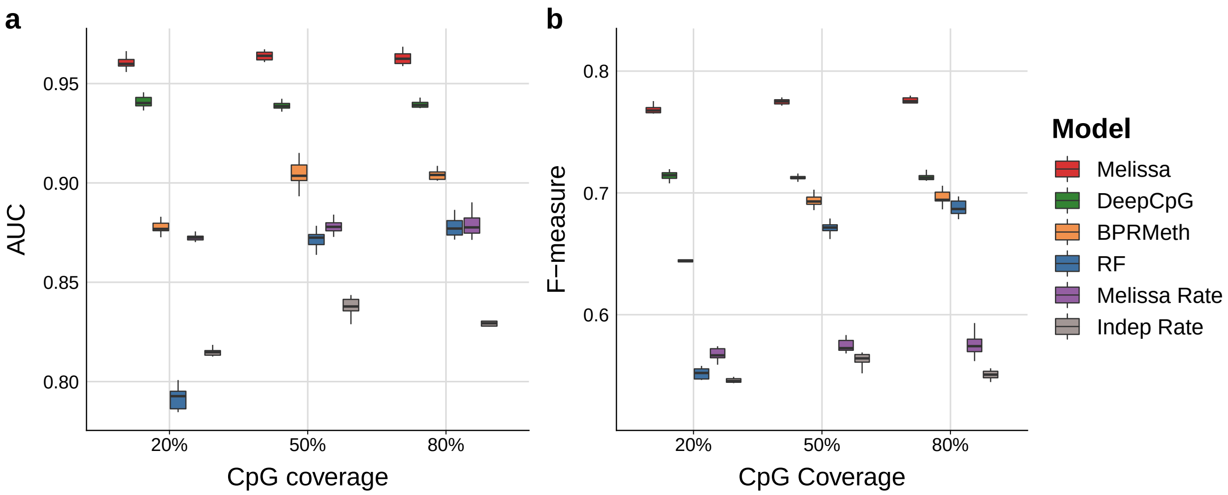

Figure S4: Melissa robustly imputes CpG methylation states on the subsampled ENCODE RRBS methylation data. Imputation performance in terms of (**a**) AUC and (**b**) F-measure for varying levels of CpG coverage for pre-defined ±2.5 kb regions around TSS. For each CpG coverage setting a total of 10 random splits of the data to training and test sets was performed. Each dot corresponds to a different simulation.

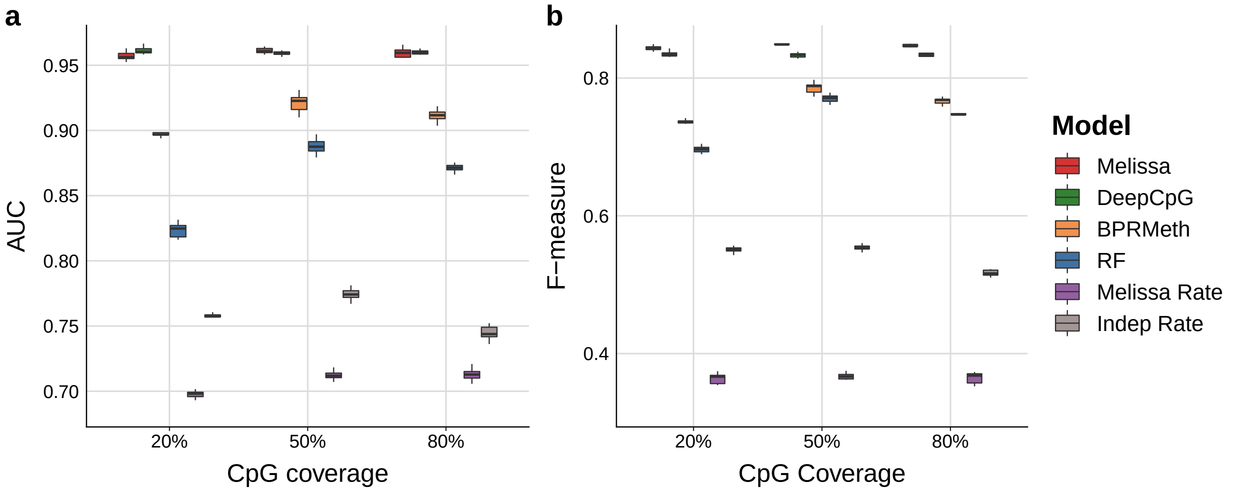

Figure S5: Melissa robustly imputes CpG methylation states on the subsampled ENCODE WGBS methylation data. Imputation performance in terms of (**a**) AUC and (**b**) F-measure for varying levels of CpG coverage for pre-defined ±2.5 kb regions around TSS. For each CpG coverage setting a total of 10 random splits of the data to training and test sets was performed. Each dot corresponds to a different simulation.

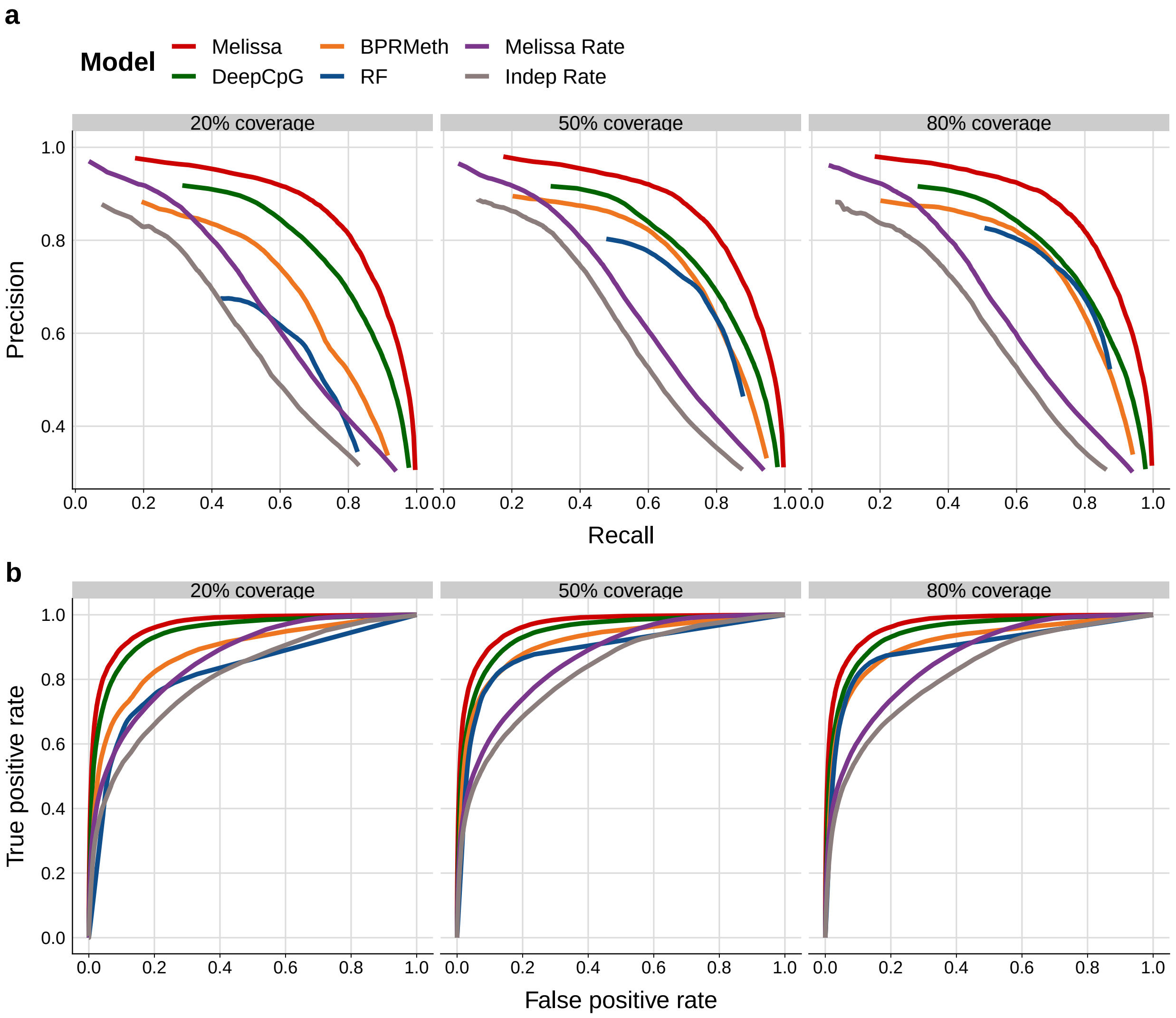

Figure S6: (**a**) Precision recall curves and (**b**) receiver operating characteristic curves on varying CpG coverage levels for imputing CpG methylation states for the subsampled ENCODE RRBS methylation data for pre-defined ±2.5 kb regions around TSS.

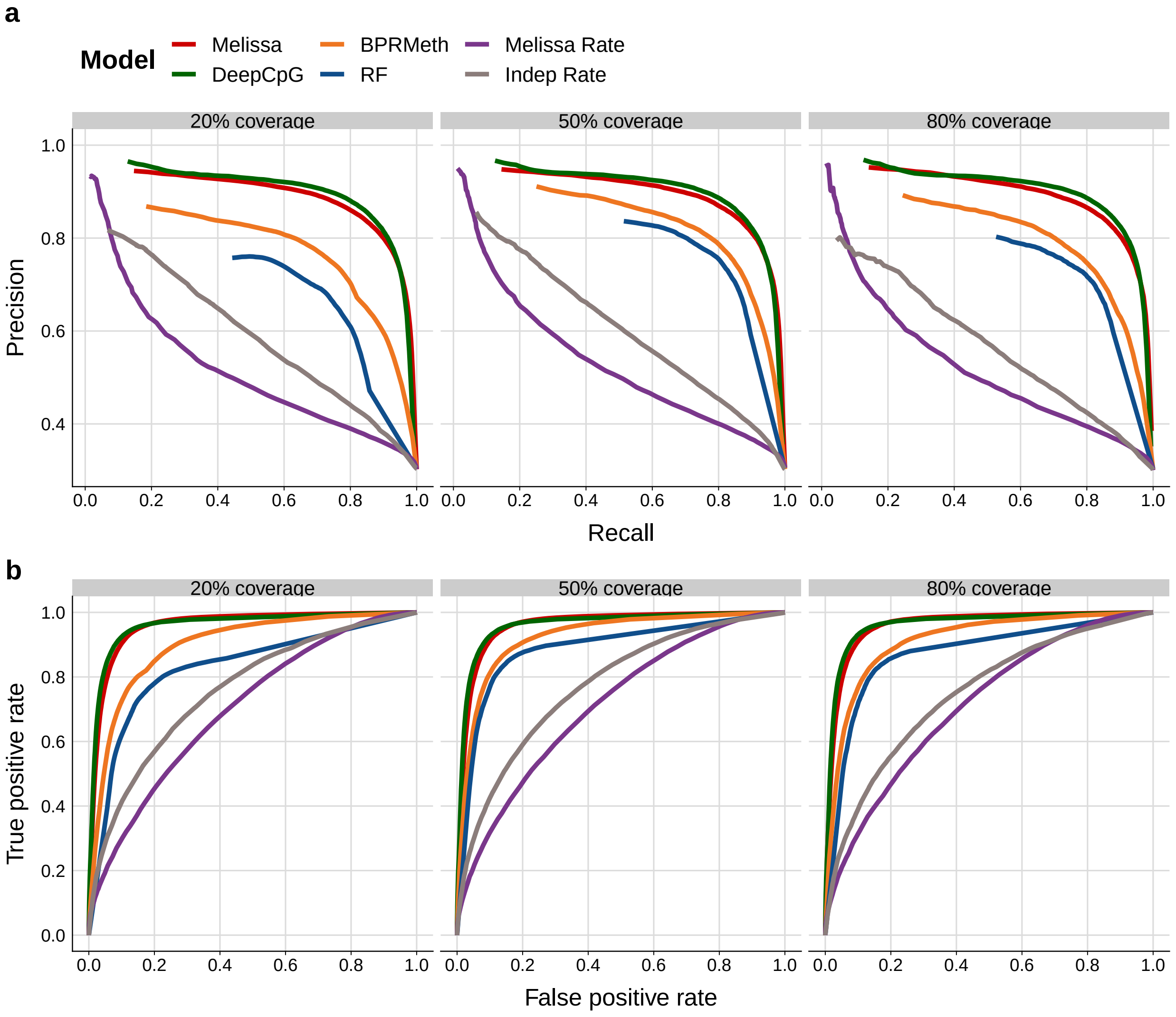

Figure S7: (**a**) Precision recall curves and (**b**) receiver operating characteristic curves on varying CpG coverage levels for imputing CpG methylation states for the subsampled ENCODE WGBS methylation data for pre-defined ±2.5 kb regions around TSS.

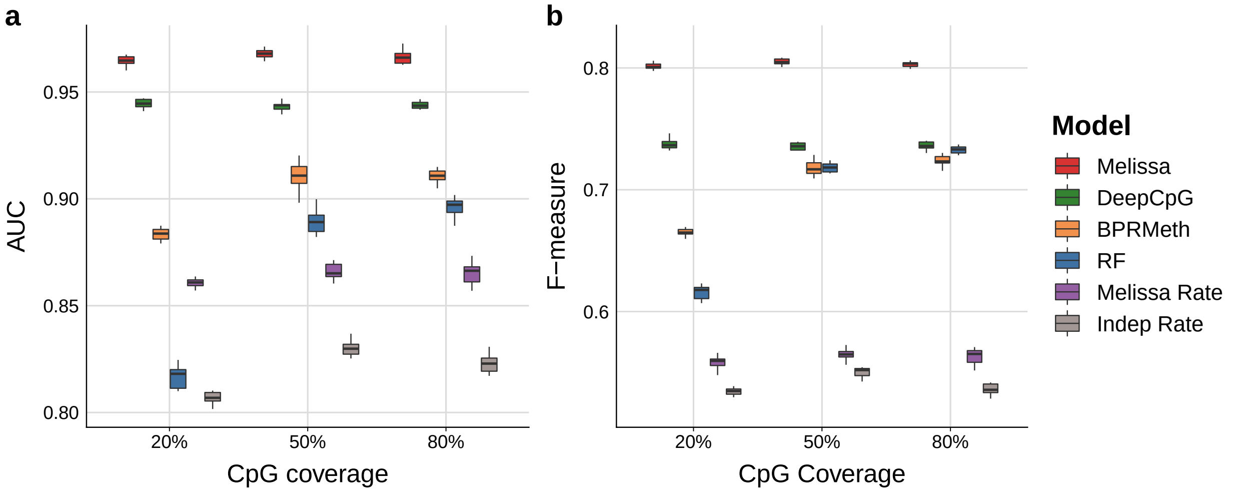

Figure S8: Melissa robustly imputes CpG methylation states on the subsampled ENCODE RRBS methylation data. Imputation performance in terms of (**a**) AUC and (**b**) F-measure for varying levels of CpG coverage for pre-defined ±5 kb regions around TSS. For each CpG coverage setting a total of 10 random splits of the data to training and test sets was performed. Each dot corresponds to a different simulation.

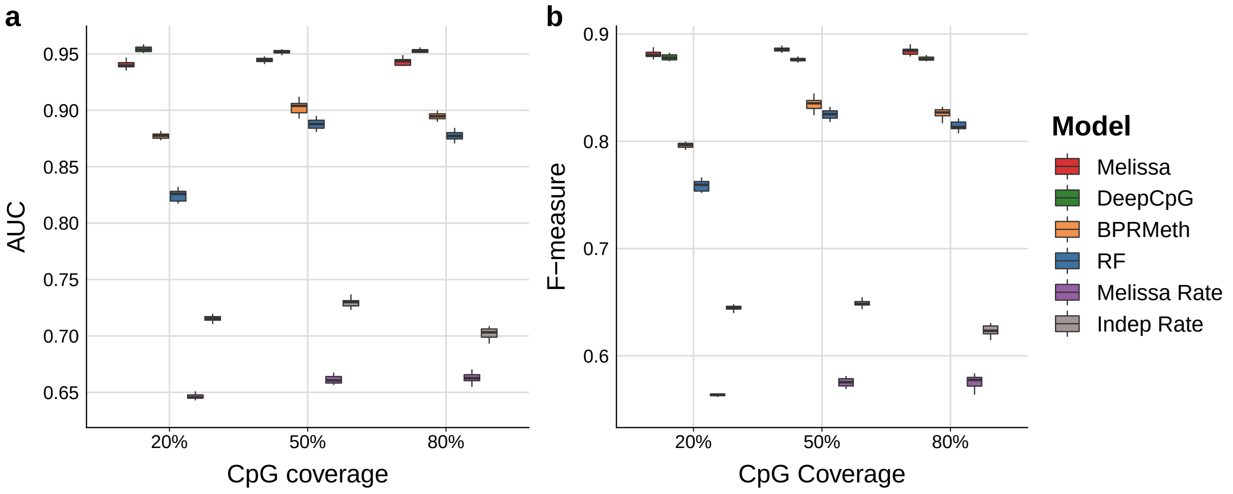

Figure S9: Melissa robustly imputes CpG methylation states on the subsampled ENCODE WGBS methylation data. Imputation performance in terms of (**a**) AUC and (**b**) F-measure for varying levels of CpG coverage for pre-defined ±5 kb regions around TSS. For each CpG coverage setting a total of 10 random splits of the data to training and test sets was performed. Each dot corresponds to a different simulation.

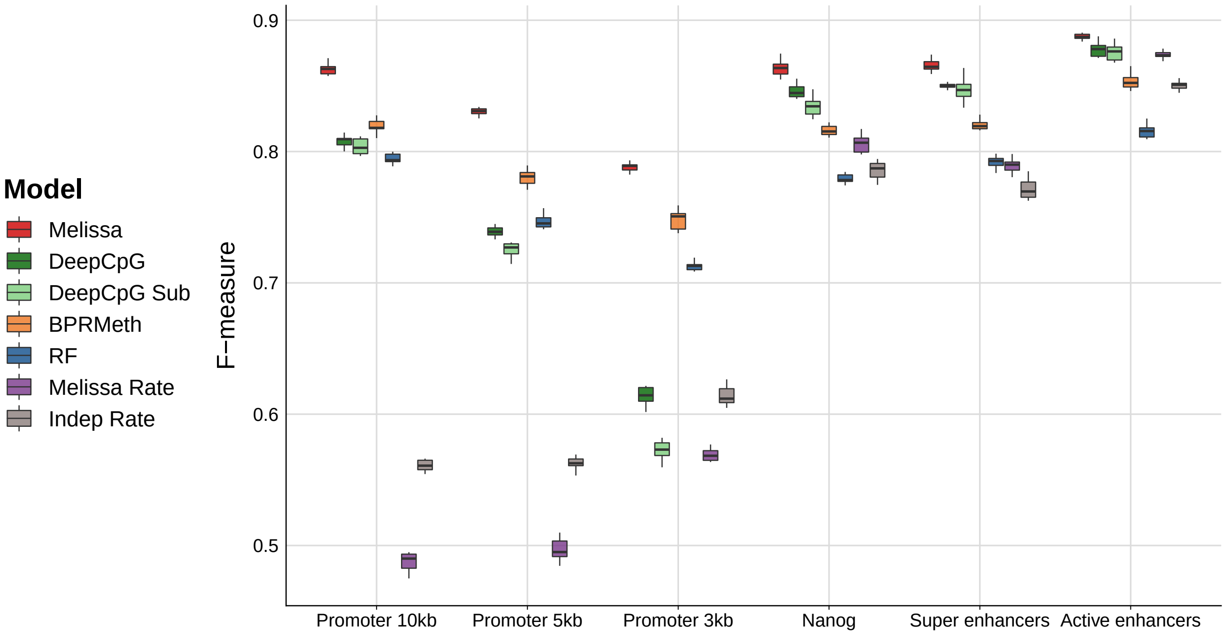

Figure S10: Prediction performance using the F-measure metric for imputing CpG methylation states of the Angermueller *et al.* (2016) dataset. Higher values correspond to better imputation performance. Each coloured boxplot indicates the performance using 10 random splits of the data in training and test sets; due to high computational costs, DeepCpG was trained only once and the boxplots denote the variability across ten random subsamplings of the test set. Shown is the prediction performance for alternative genomic contexts: promoters (±1.5 kb, ±2.5 kb and ±5 kb regions), active enhancers, super enhancers and Nanog regulatory regions.

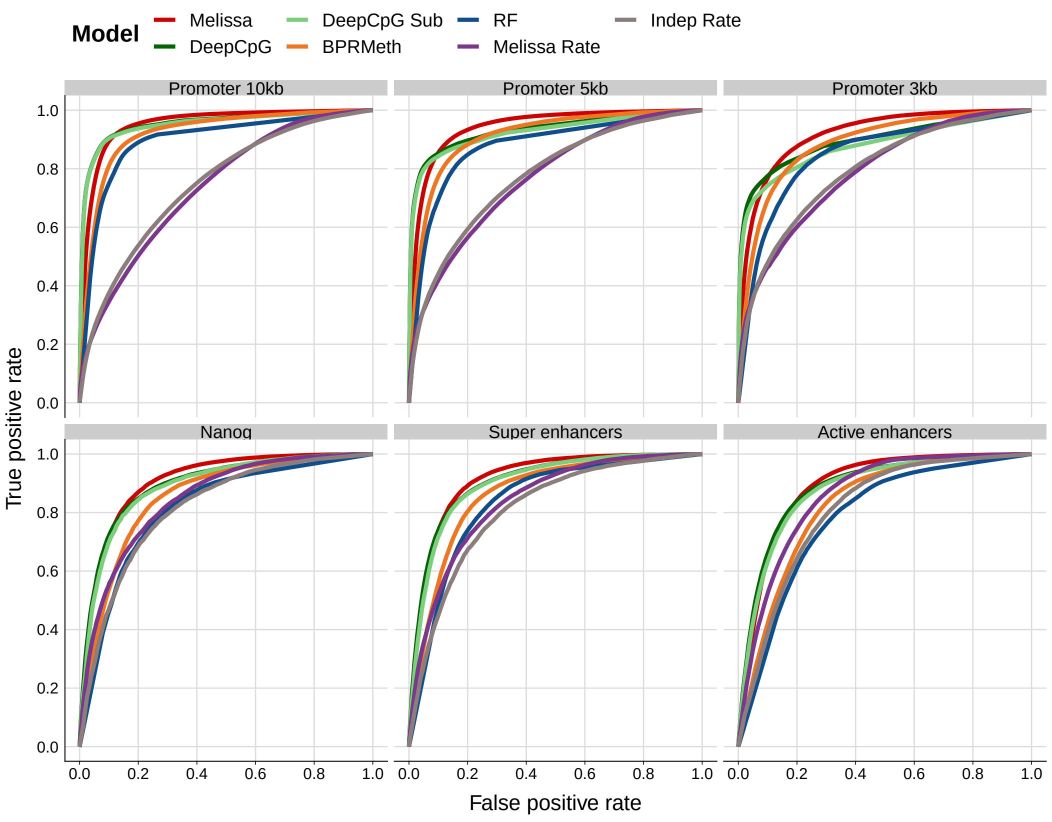

Figure S11: Receiver operating characteristic curves for imputing CpG methylation states of the Angermueller *et al.* (2016) dataset.

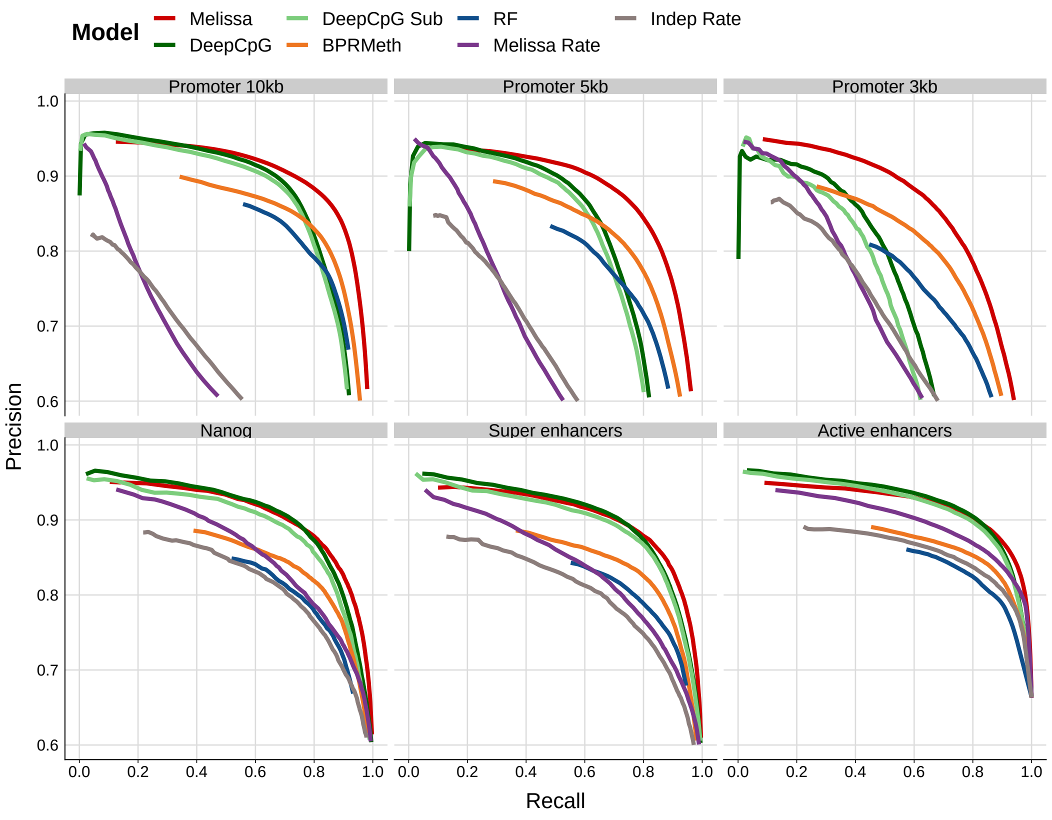

Figure S12: Precision recall curves for imputing CpG methylation states of the Angermueller *et al.* (2016) dataset.

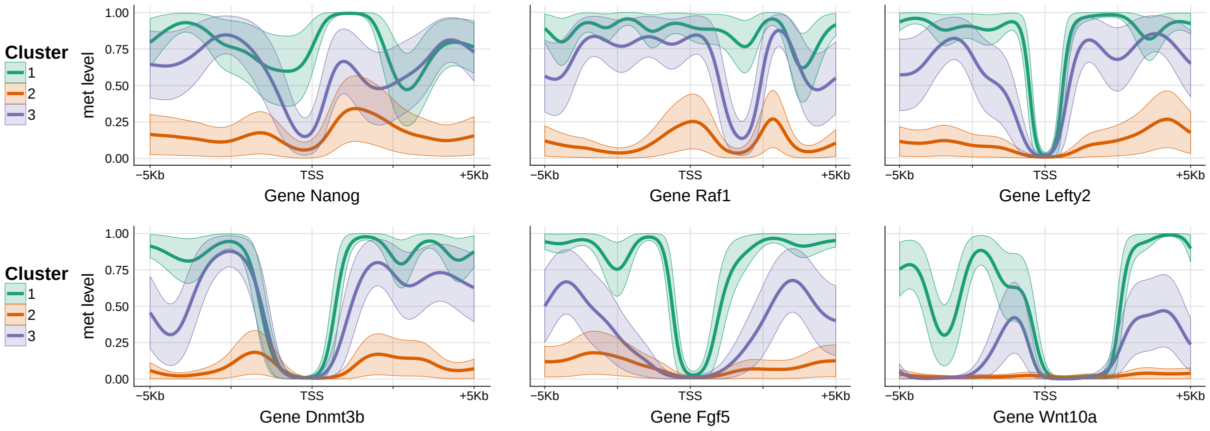

Figure S13: Example profiles for different promoter regions of developmental genes with window length ±5kb for the Angermueller *et al.* (2016) dataset. Melissa identified three cell sub-populations.

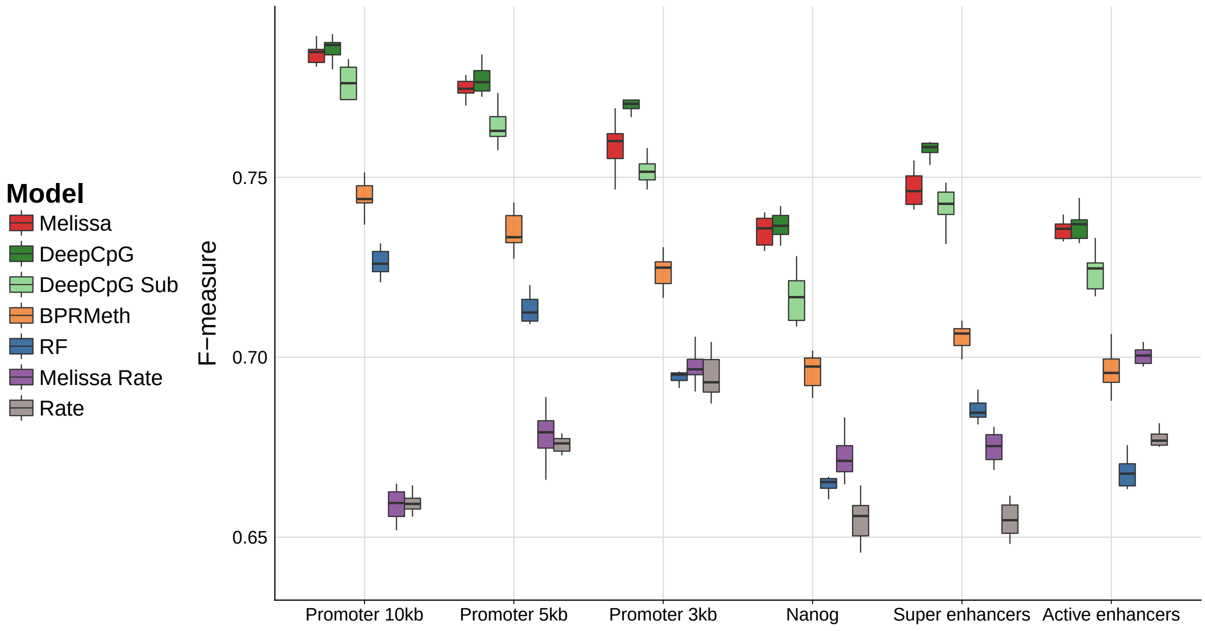

Figure S14: Prediction performance using the F-measure metric for imputing CpG methylation states of the Smallwood *et al.* (2014) dataset. Higher values correspond to better imputation performance. Each coloured boxplot indicates the performance using 10 random splits of the data in training and test sets; due to high computational costs, DeepCpG was trained only once and the boxplots denote the variability across ten random subsamplings of the test set. Shown is the prediction performance for alternative genomic contexts: promoters (±1.5 kb, ±2.5 kb and ±5 kb regions), active enhancers, super enhancers and Nanog regulatory regions.

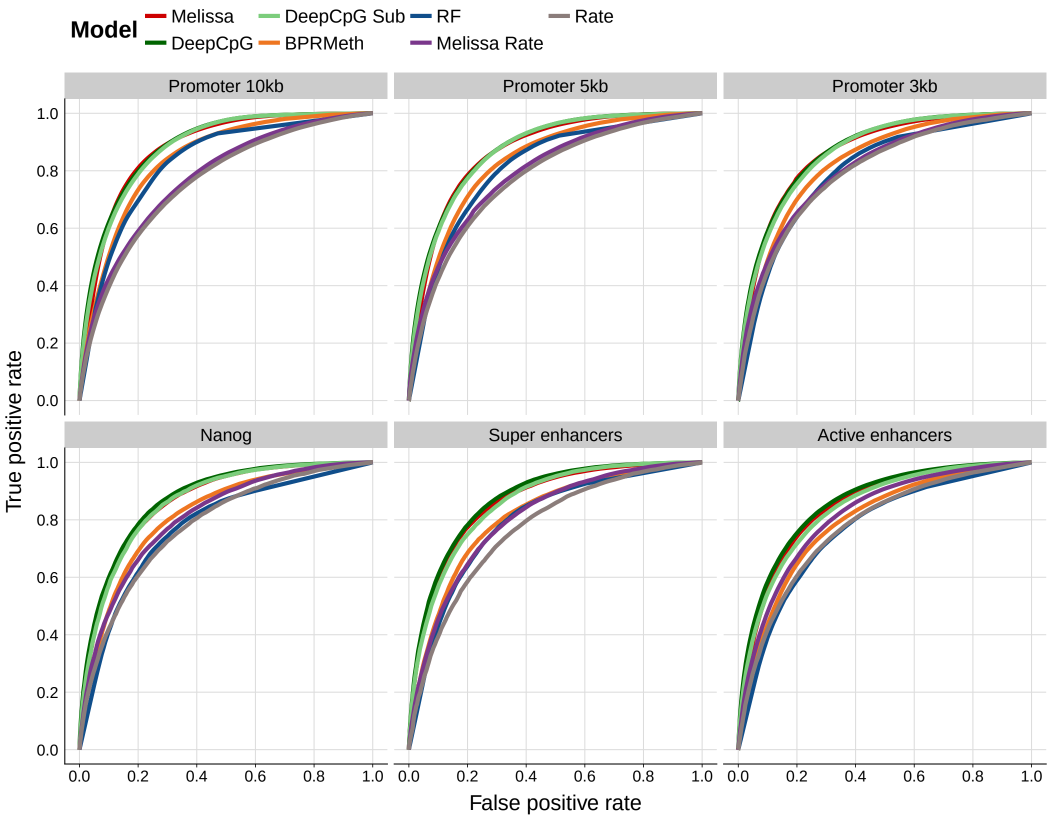

Figure S15: Receiver operating characteristic curves for imputing CpG methylation states of the Smallwood *et al.* (2014) dataset.

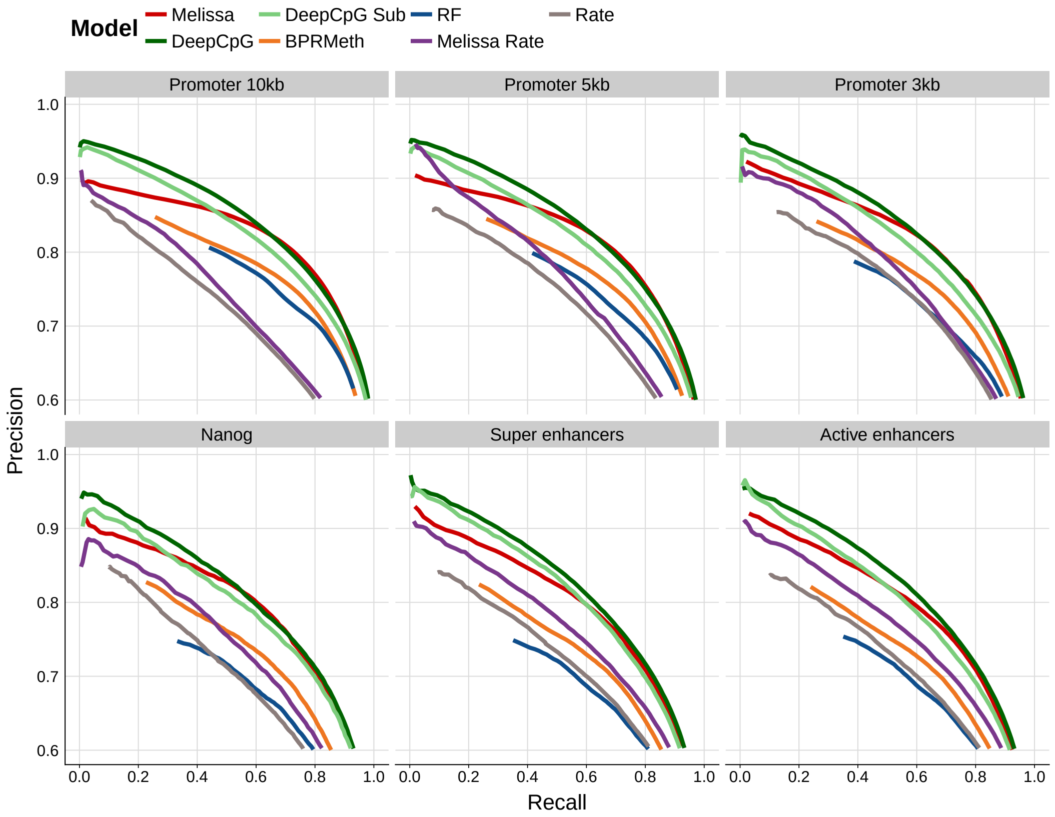

Figure S16: Precision recall curves for imputing CpG methylation states of the Smallwood *et al.* (2014) dataset.

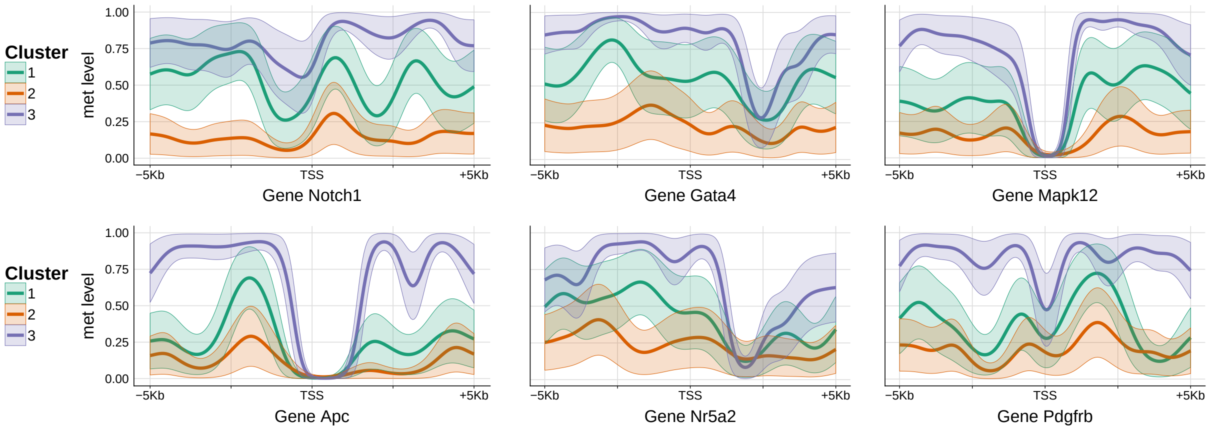

Figure S17: Example profiles for different promoter regions with window length ±5 kb for the Smallwood *et al.* (2014) dataset. Melissa identified three cell sub-populations.

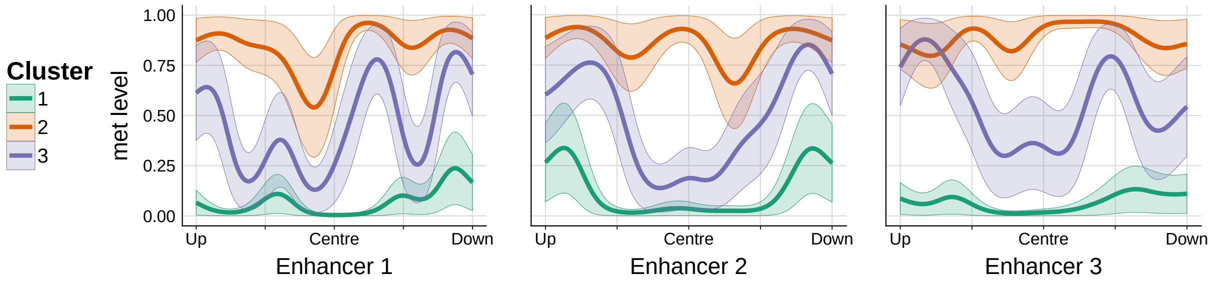

Figure S18: Example profiles for different enhancer regions for the Smallwood *et al.* (2014) dataset. Melissa identified three cell sub-populations.

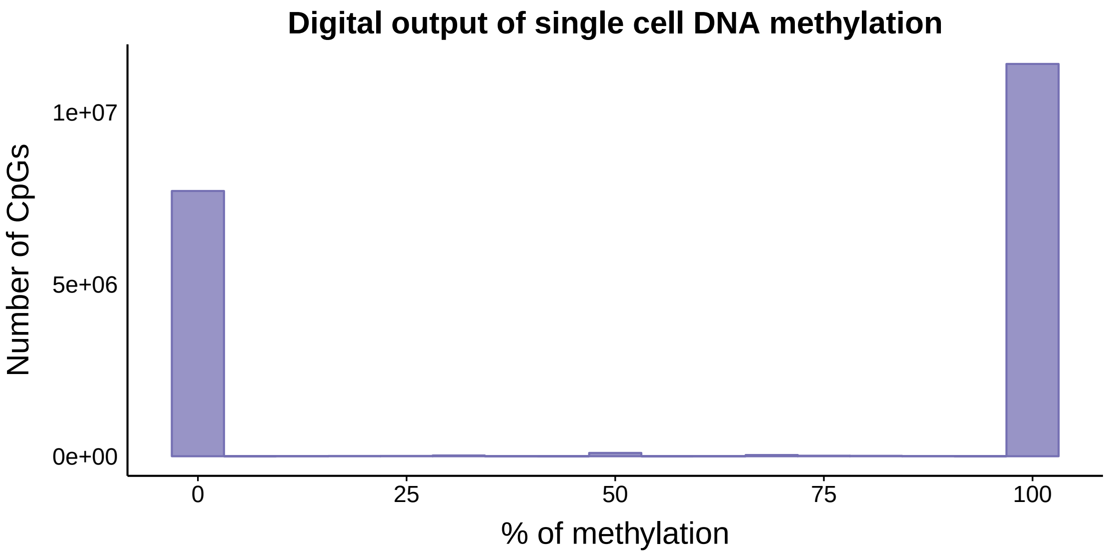

Figure S19: Digital output of single cell DNA methylation. Histogram of the distribution of CpG methylation values for 10 randomly sampled single cells from the Angermueller *et al.* (2016) study. As expected, the proportion of binary CpGs is very high (around 98.8%) and only around 0.5% of CpG sites are hemi-methylated.

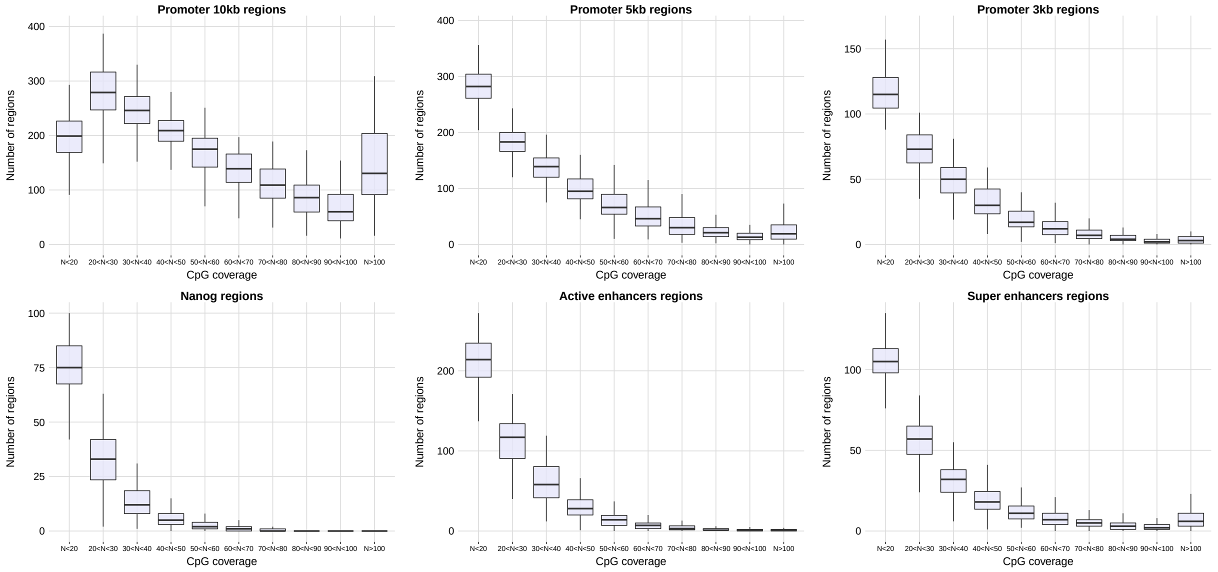

Figure S20: Boxplots of CpG coverage distribution across different genomic contexts for the Angermueller *et al.* (2016) dataset after the filtering process. The x-axis shows CpG coverage bins and the y-axis shows the distribution of the number of genomic regions with N CpGs covered across cells, that is, each dot in the boxplot represents a different cell.

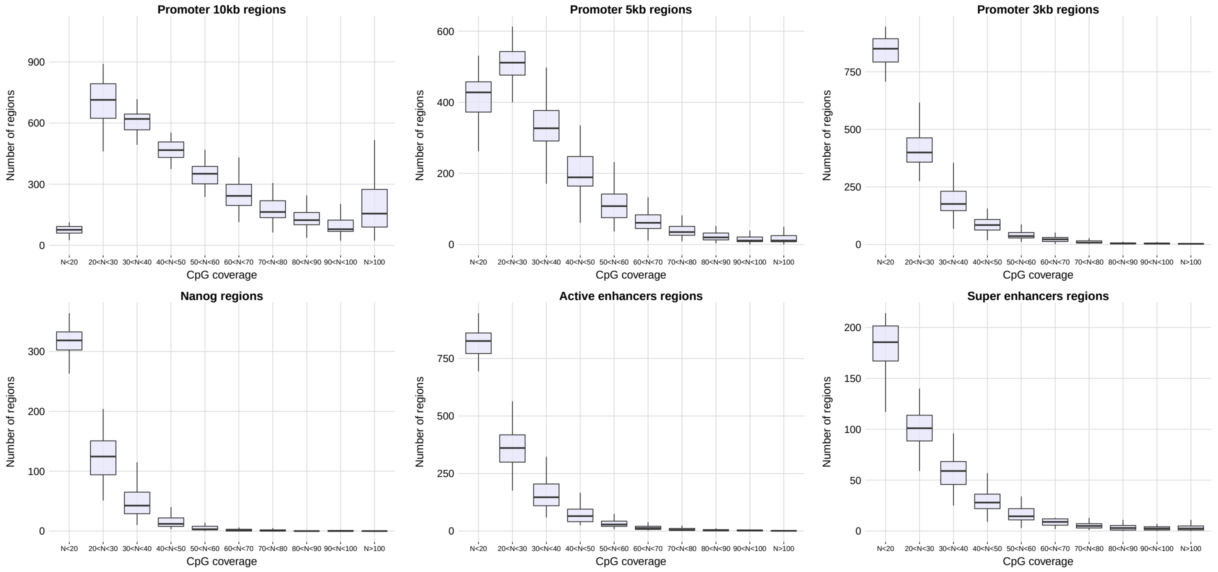

Figure S21: Boxplots of CpG coverage distribution across different genomic contexts for the Smallwood *et al.* (2014) dataset after the filtering process. The x-axis shows CpG coverage bins and the y-axis shows the distribution of the number of genomic regions with N CpGs covered across cells, that is, each dot in the boxplot represents a different cell.

3 Additional tables

Genomic context	CpGs (in millions)	Time (in hours)
Promoter 10kb	6	5.6
Promoter 5kb	2.1	2.9
Promoter 3kb	0.62	1.31
Nanog	0.18	0.61
Super enhancers	0.5	1
Active enhancers	0.7	1.76

Table S1: Melissa training time for the Angermueller *et al.* (2016) mouse ESC dataset. Across different genomic contexts are shown the total number of CpGs used for training set and the time required (in hours) for running Melissa to impute and cluster single cells. Note that the numbers in millions refer to the total number of CpGs not the genomic regions which are generally up to thousands. As a comparison, the DeepCpG model took about three to four days to train on around four million CpGs.

Genomic context	CpGs (in millions)	Time (in hours)
Promoter 10kb	4.13	4
Promoter 5kb	1.54	2.21
Promoter 3kb	0.98	1.83
Nanog	0.26	0.9
Super enhancers	0.29	0.9
Active enhancers	0.85	2

Table S2: Melissa training time for the Smallwood *et al.* (2014) mouse ESC dataset. Across different genomic contexts are shown the total number of CpGs used for training set and the time required (in hours) for running Melissa to impute and cluster single cells. Note that the numbers in millions refer to the total number of CpGs not the genomic regions which are generally up to thousands. As a comparison, the DeepCpG model took about three to four days to train on around four million CpGs.

Genomic context	Smallwood study	Angermueller study
Promoter 10kb	21%	17%
Promoter 5kb	23%	20%
Promoter 3kb	24%	24%
Nanog	19%	17%
Super enhancers	19%	12%
Active enhancers	25%	17%

Table S3: Sparsity level of the two scBS-seq data after filtering across different genomic regions.

References

Angermueller, C., Clark, S. J., Lee, H. J., Macaulay, I. C., Teng, M. J., Hu, T. X., Krueger, F., Smallwood, S. A., Ponting, C. P., Voet, T., Kelsey, G., Stegle, O., and Reik, W. (2016). Parallel single-cell sequencing links transcriptional and epigenetic heterogeneity. *Nature methods*, **13**(3), 229–32.

Blei, D. M., Kucukelbir, A., and McAuliffe, J. D. (2017). Variational Inference: A Review for Statisticians. *Journal of the American Statistical Association*, **112**(518), 859–877.

Smallwood, S. a., Lee, H. J., Angermueller, C., Krueger, F., Saadeh, H., Peat, J., Andrews, S. R., Stegle, O., Reik, W., and Kelsey, G. (2014). Single-cell genome-wide bisulfite sequencing for assessing epigenetic heterogeneity. *Nature Methods*, **11**(8), 817–20.
